# Unraveling Prevalence and Effects of Deleterious Mutations in Maize Elite Lines across Decades of Modern Breeding

**DOI:** 10.1093/molbev/msad170

**Published:** 2023-07-26

**Authors:** Shichao Sun, Baobao Wang, Changyu Li, Gen Xu, Jinliang Yang, Matthew B Hufford, Jeffrey Ross-Ibarra, Haiyang Wang, Li Wang

**Affiliations:** Shenzhen Branch, Guangdong Laboratory for Lingnan Modern Agriculture, Genome Analysis Laboratory of the Ministry of Agriculture, Agricultural Genomics Institute at Shenzhen, Chinese Academy of Agricultural Sciences, Shenzhen, China; Biotechnology Research Institute, Chinese Academy of Agricultural Sciences, Beijing, China; Biotechnology Research Institute, Chinese Academy of Agricultural Sciences, Beijing, China; Department of Agronomy and Horticulture, University of Nebraska-Lincoln, Lincoln, NE, USA; Department of Agronomy and Horticulture, University of Nebraska-Lincoln, Lincoln, NE, USA; Department of Ecology, Evolution, and Organismal Biology, Iowa State University, Ames, IA, USA; Department of Evolution and Ecology, University of California, Davis, CA, USA; State Key Laboratory for Conservation and Utilization of Subtropical Agro-Bioresources, South China Agricultural University, Guangzhou, China; Guangdong Laboratory for Lingnan Modern Agriculture, Guangzhou, China; Shenzhen Branch, Guangdong Laboratory for Lingnan Modern Agriculture, Genome Analysis Laboratory of the Ministry of Agriculture, Agricultural Genomics Institute at Shenzhen, Chinese Academy of Agricultural Sciences, Shenzhen, China; Department of Ecology, Evolution, and Organismal Biology, Iowa State University, Ames, IA, USA; Kunpeng Institute of Modern Agriculture at Foshan, Chinese Academy of Agricultural Sciences, Foshan, China

**Keywords:** deleterious mutation, gene expression, heterosis, purifying selection, SNP effect size

## Abstract

Future breeding is likely to involve the detection and removal of deleterious alleles, which are mutations that negatively affect crop fitness. However, little is known about the prevalence of such mutations and their effects on phenotypic traits in the context of modern crop breeding. To address this, we examined the number and frequency of deleterious mutations in 350 elite maize inbred lines developed over the past few decades in China and the United States. Our findings reveal an accumulation of weakly deleterious mutations and a decrease in strongly deleterious mutations, indicating the dominant effects of genetic drift and purifying selection for the two types of mutations, respectively. We also discovered that slightly deleterious mutations, when at lower frequencies, were more likely to be heterozygous in the developed hybrids. This is consistent with complementation as a potential explanation for heterosis. Subsequently, we found that deleterious mutations accounted for more of the variation in phenotypic traits than nondeleterious mutations with matched minor allele frequencies, especially for traits related to leaf angle and flowering time. Moreover, we detected fewer deleterious mutations in the promoter and gene body regions of differentially expressed genes across breeding eras than in nondifferentially expressed genes. Overall, our results provide a comprehensive assessment of the prevalence and impact of deleterious mutations in modern maize breeding and establish a useful baseline for future maize improvement efforts.

## Introduction

In each generation, all organisms exhibit stacks of mutations, the majority of which are deleterious and decrease the fitness of individuals (Eyre-Walker and Keightley 2007). The term “mutation load” was originally used to describe this reduction in fitness due to deleterious mutations (Haldane 1937; Muller 1950; Kono et al. 2016). It is calculated as the proportional reduction in average individual fitness compared with the maximum fitness, using the formula (*W*_max_ − *W*_ave_)/*W*_max_ (Crow 1989). However, this concept is artificial, as maximum fitness is a hypothetical construct, limiting the study of mutation load to a theoretical framework with very few empirical cases. Recently, there has been a surge in studies on mutation load, primarily due to the availability of large-scale population resequencing data and the development of population genetic summary statistics as proxies for mutation load (Simons and Sella 2016; Kremling et al. 2018; Lian et al. 2019; Kutschera et al. 2020; Rougemont et al. 2020; Lozano et al. 2021). These advances have enabled researchers to estimate mutation load in natural populations, providing insights into the evolutionary mechanisms shaping genetic variation and the genetic basis of complex traits.

Population genetic theories suggest that the burden of deleterious mutations is influenced by multiple factors, including demographic history, selection, and mating system. A shift in the effective population size (*N_e_*) can affect the efficiency of purifying selection and genetic drift, both of which can impact the prevalence of deleterious mutations in a population (Slatkin and Excoffier 2012; Fu et al. 2014; Henn et al. 2015; Marsden et al. 2016; Simons and Sella 2016; Zhang et al. 2016). Simulations (Kim et al. 2018) and empirical evidence (Wang et al. 2017; Liu et al. 2022) show that gene flow from a larger population can reduce the genetic load of a smaller receiving population by carrying fewer deleterious mutations. Furthermore, the mating system plays a prominent role in determining the genetic load. Inbreeding may have opposite effects on deleterious mutations (Glémin 2003; Gaut et al. 2015; Moyers et al. 2018; Zeitler et al. 2020). On one hand, it can expose recessive deleterious alleles to purifying selection, thereby reducing the mutation load (Robinson et al. 2018; Lozano et al. 2021). On the other hand, inbreeding can decrease the effective population size and recombination, increasing genetic drift and reducing the efficacy of selection, potentially increasing the mutation load (Nordborg 2000).

Evolutionary forces play a crucial role in crop domestication and improvement. The domestication bottleneck, which refers to the reduction of genetic diversity and effective population size, increases the burden of deleterious mutations in several crops (Lu et al. 2006; Liu et al. 2017; Ramu et al. 2017; Wang et al. 2017; Zhang et al. 2019; Zhang et al. 2021), known as the “domestication cost” (Gaut et al. 2018). Recent studies on maize suggest that large-effect deleterious mutations are purged during domestication, while small-effect deleterious variants are the main contributors to inbreeding depression and polygenic load (Samayoa et al. 2021). However, the shift from the dominant outcrossing in wild relatives to inbreeding in domesticates has reduced the count of deleterious mutations in sorghum, which deviates from the “domestication cost” hypothesis (Lozano et al. 2021). Taken together, previous studies imply that the evolutionary history of crops largely affects the pattern and dynamics of deleterious mutations, posing challenges for the molecular breeding of elite cultivars, particularly in eliminating deleterious mutations.

The role of deleterious mutations in shaping phenotypic variation has been widely reported (Yang et al. 2017; Ramstein et al. 2020; Samayoa et al. 2021; Ramstein and Buckler 2022), but their contribution to heterosis (hybrid vigor), a landmark innovation in modern agriculture, is not well understood (Springer and Stupar 2007). Large-effect deleterious alleles may contribute less to phenotypic variance than small-effect deleterious alleles, owing to their low frequency in the population, whereas small-effect deleterious alleles are likely to impact heterosis (Ramstein et al. 2020). Three hypotheses have been proposed to explain the causes of heterosis: dominance effect (interaction of alleles within a locus), epistasis (interaction of alleles among loci), and overdominance (selection for heterozygous genotypes) (Springer and Stupar 2007; Hochholdinger and Baldauf 2018). The dominance model suggests that the slightly deleterious recessive alleles in many genes are complemented by superior dominant alleles in the hybrids. Previous studies have shown that incomplete dominance contributes to heterosis in maize (Yang et al. 2017). Xiao et al. (2021) demonstrated how deleterious epistatic interactions in maize parents were complemented in hybrids, emphasizing the significance of both epistasis and dominance models. A recent study revealed the overdominant effect of an important ethylene biosynthesis gene, *ZmACO2*, located within the major quantitative trait locus (QTL) for ear length and kernel yield, in promoting heterosis (Wang et al. 2023). However, a comprehensive atlas comparing the deleterious alleles between hybrids and their parents and their contributions to heterosis has not been created. Therefore, a further understanding of the dynamics of deleterious mutations in the process of hybridization could be incorporated, ensuring sustainable crop yields.

Maize (*Zea mays* L.) is one of the main cereal sources for human food and animal feed and is a model plant for studying various aspects of plant biology, such as domestication, population expansion, local adaptation, and genomic selection (Romay et al. 2013). Since the discovery of inbreeding and heterosis in maize by Shull (1908) and East (1908) at the beginning of the 20th century, maize genetics and breeding communities worldwide have developed diverse genetic materials and advanced breeding lines. Previous studies have investigated the fluctuations in deleterious mutations and have linked the increase of such mutations to domestication and expansion, primarily due to the bottlenecks and genetic drift (Wang et al. 2017). This contrasts with the results obtained in sunflowers, where no significant change in harmful mutations was observed during improvement (Renaut and Rieseberg 2015), but is consistent with the decreased mutation load during improvement in barley (Kono et al. 2019). Yang et al. (2017) reported that improved cultivars showed a decline in deleterious mutations compared with landraces. However, previous reports have focused on the dynamics of deleterious mutations between inbred lines (ILs) and landraces in maize, overlooking the prevalence of deleterious mutations with varying degrees of severity throughout the ongoing process of breeding ILs.

In this study, the published whole-genome resequencing data of 163 and 187 maize ILs from the United States and China, respectively, were utilized. Specifically, the US lines consist of two groups (Public_US before 2003 and expired Plant Variety Protection Act certificates [Ex_PVP] after 2003), whereas the Chinese lines consist of three groups (ILs used for single-cross hybrids and released in the 1960s and 1970s, 1980s and 1990s, 2000s and 2010s respectively). Additionally, 120 hybrids, which were historically commercial varieties in the Chinese maize seed market and were derived from crossing subsets of 350 ILs, were also included in the analysis. The aim of this study was to examine how the prevalence of deleterious mutations contributes to heterosis in maize and to answer the following questions: 1) What is the dynamic pattern of genetic load in maize over decades of breeding efforts? 2) How does complementation play a role in hybrid vigor with regard to deleterious mutations? 3) Do deleterious mutations affect phenotypic traits? If so, to what extent? The study proposed a comprehensive framework for the dynamics of deleterious mutations in the process of maize improvement and provided new insights into future breeding strategies.

## Results

### Percentage of Heterosis of Agronomic Traits over Breeding Eras

**Fig. 1.**
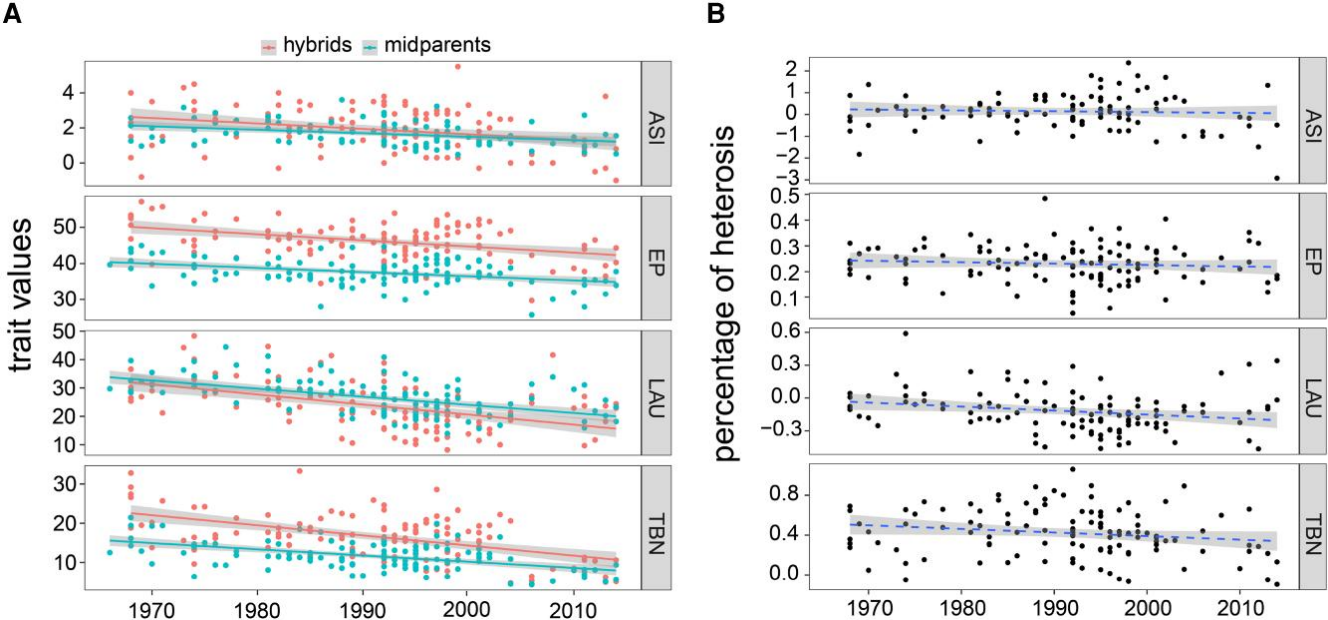
Trends of phenotypic traits in 120 hybrids over breeding eras. (*A*) Changes in key agronomic traits, including the shortened ASI, reduced relative EH (EP), LAU, and TBN, were observed in 120 hybrids over the course of the breeding eras. (*B*) The percentage of heterosis for ASI, EP, LAU, and TBN in 120 hybrids. The solid line indicates a significant correlation, while the dashed line represents a nonsignificant relationship. The shaded area denotes the 95% confidence interval.

The percentage of heterosis for agronomic traits has been a topic of interest for maize breeders over the course of the breeding eras (Duvick and Cassman 1999; Duvick 2005; Troyer and Wellin 2009). Maize yield has increased by more than seven times since the first introduction of inbreeding and heterosis in maize breeding (East 1908; Shull 1908). In this study, we evaluated changes in 12 key agronomic traits over time in 120 derived hybrids and compared them with trends in their parental ILs (Wang et al. 2020). The hybrids were selected from historically commercial varieties, widely available in Chinese maize seed market, and were regenerated by crossing subsets of 350 ILs based on known pedigree information (supplementary table S1, Supplementary Material online). Our findings showed a significant decrease in the anthesis to silking interval (ASI; *P* = 0.002), relative ear height (ear height/plant height [EP]; P = 7.38e−06), tassel branch number (TBN; *P* = 2.80e−09), and upper leaf angle (LAU; *P* = 4.51e−08) in the 120 hybrids over the course of the breeding eras. These changes were consistent with the direction of selection for the same traits in the parental ILs (fig. 1*A*; supplementary fig. S1, Supplementary Material online), indicating that the improvement in traits in hybrids was consistent with that in the parental ILs. Moreover, the reduction in the abovementioned four traits in the hybrids was similar to those of the midparents across breeding eras (supplementary table S2, Supplementary Material online). However, there was no significant change observed in heterosis or the percentage of heterosis, i.e., (performance of hybrid − performance of midparent)/performance of midparent (*P* > 0.01; fig. 1*B*), indicating that the performance of hybrids was consistent with that of the parental ILs.

### Prevalence of Deleterious Mutations over Breeding Eras

**Fig. 2.**
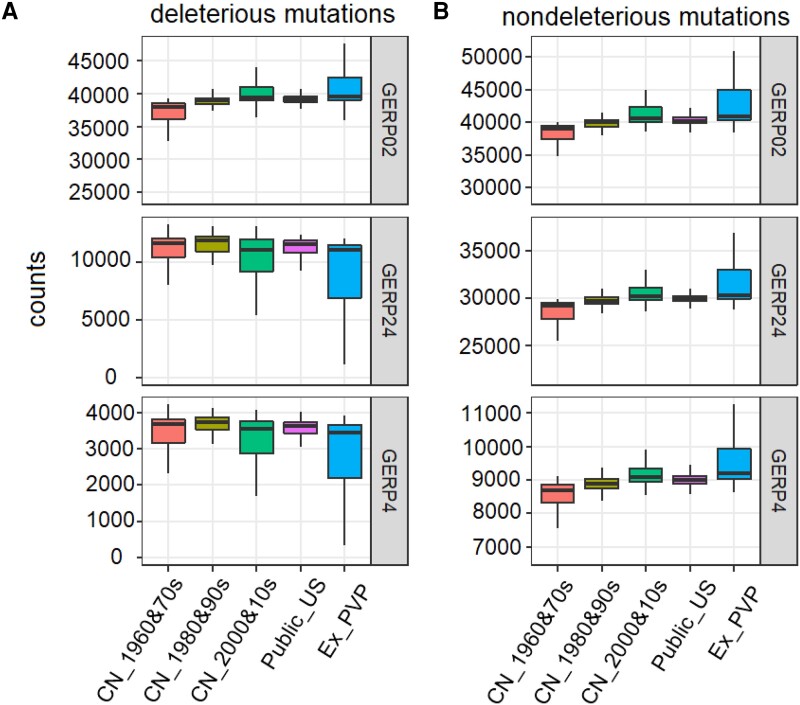
Count of homozygous deleterious (*A*) and nondeleterious (*B*) alleles in ILs from stratified eras in China and the United States. The allele frequencies of randomly selected nondeleterious mutations were matched with those of deleterious mutations. “Nondeleterious” indicates SNPs with GERP ≤ 0, GERP02 represents the slightly deleterious mutations, GERP24 indicates the moderately deleterious mutations, and GERP4 implies the strongly deleterious mutations. CN_1960&70s, CN_1980&90s, and CN_2000&10s represent Chinese maize ILs collected from three breeding eras in China: the 1960s and 1970s, the 1980s and 1990s, and the 2000s and 2010s, respectively. Public_US and Ex-PVP represent the US maize ILs collected during two breeding eras: before 2003 and after 2003. Each box represents the median and interquartile range (IQR). The whiskers represent the range of 1.5 times IQR.

The absence of an increase in heterosis and the percentage of heterosis prompted us to investigate how the burden of deleterious mutations fluctuated in both ILs and hybrids during breeding eras. To explore the prevalence of deleterious mutations in the maize improvement process, we computed the number of deleterious mutations in each population that represented the stratified breeding eras in China and the United States. Using Genomic Evolutionary Rate Profiling (GERP) to identify putatively deleterious mutations (Cooper et al. 2005), we calculated the number of derived deleterious alleles per genome under both additive and recessive modes across the three levels of deleterious mutation severity. Interestingly, in Chinese ILs, slightly deleterious alleles (0 < GERP ≤ 2) accumulated across breeding eras under both additive (1.9–2.2% more; *P* < 0.0003, Wilcoxon test; supplementary fig. S2, Supplementary Material online) and recessive (2.9–5.7% more; *P* < 0.0003, Wilcoxon test; fig. 2*A*) modes, consistent with the trend observed for nondeleterious mutations (fig. 2*B*). This suggests nearly neutral characteristics of slightly deleterious mutations and possibly relaxed purifying selection in purging slightly deleterious alleles. However, the count of moderately deleterious alleles (2 < GERP ≤ 4) decreased from 1980s and 1990s ILs to 2000s and 2010s ILs under both additive (8.3% less; *P* = 0.0007, Wilcoxon test; supplementary fig. S2, Supplementary Material online) and recessive (7.4% less; *P* = 0.0036, Wilcoxon test; fig. 2*A*) modes, contrasting with the dynamics of nondeleterious mutations (fig. 2*B*). This points to the continuous purging (i.e., increased purifying selection) of moderately deleterious mutations owing to the elevated level of inbreeding (supplementary fig. S3, Supplementary Material online; inbreeding coefficient: CN1 [Chinese ILs developed in 1960s and 1970s] < CN2 [1980s and 1990s] < CN3 [2000s and 2010s] and Public_US < Ex-PVP; runs of homozygosity [ROH]: CN1 < CN2 < CN3 and Public_US < Ex-PVP). Similarly, the strongly deleterious alleles (GERP > 4) were also purged out through continuous breeding efforts under both recessive (8.0% less; *P* = 0.0025, Wilcoxon test; fig. 2*A*) and additive modes (8.8% less; *P* = 0.0003, Wilcoxon test; supplementary fig. S2, Supplementary Material online). The number of moderately and strongly deleterious mutations did not change from the 1960s and 1970s to the 1980s and 1990s Chinese ILs (*P* > 0.66). This likely reflects the stagnant breeding process during those years in China. Among the US ILs, Ex-PVP ILs had higher levels of the slightly deleterious mutations than Public_US ILs, with 3.8% more in the additive mode (*P* = 1.1e−06, Wilcoxon test) and 4.0% more in the recessive mode (*P* = 0.0001), concordant with the dynamics of nondeleterious mutations (fig. 2). In contrast, moderately and strongly deleterious mutations were largely purged out from the genetic pools of all ILs (16.5–17.0% less, *P* < 0.0003, Wilcoxon test; fig. 2*A*; supplementary fig. S2, Supplementary Material online). The higher percentage of reduction in moderately and strongly deleterious mutations across eras in the US ILs compared with Chinese ILs suggests that breeding in the United States is more efficient than in China. This may result from the availability of resources and the adoption of more effective methodologies. We also estimated the mutation load in modern maize populations in China and the United States using 25,339 nonsynonymous deleterious single nucleotide polymorphisms (SNPs) defined by a SIFT score of <0.05 (Vaser et al. 2016). We observed similar patterns in the mutation load among the populations as with the GERP-based estimation (supplementary fig. S4, Supplementary Material online). In addition, compared with the early ILs, more fixed loads were observed in more recent elite ILs (supplementary fig. S5, Supplementary Material online) in both China (CN_1980&90s vs. CN_1960&70s, *P* = 6.46e−11, Wilcoxon test; CN_2000&10s vs. CN_1980&90s, *P* = 6.47e−11, Wilcoxon test) and the United States (*P* ≈ 0, Wilcoxon test), consistent with the expectations based on the reduced genetic diversity and increased genetic drift in the recently ILs. Overall, slightly deleterious mutations that accumulated during the breeding process behaved as if they were almost neutral when the selection coefficient was less than the inverse of the effective population size. Moderately and strongly deleterious mutations were continuously purged from the genetic pool of ILs through purifying selection.

The fate of slightly and strongly deleterious mutations was also reflected in the site frequency spectrum (SFS). We polarized the SNPs by utilizing *Sorghum bicolor* alleles as the ancestral state and found that the derived SFS shifted to the left as the mutation effect became stronger. This resulted in more low-frequency and fewer high-frequency deleterious mutations when the evolutionary constraint intensified, suggesting an effect of increased purifying selection (supplementary fig. S6, Supplementary Material online).

We also examined how the count of deleterious alleles changed over time in 120 hybrid lines produced by crossing subsets of the 350 ILs. By examining the trend of deleterious mutations of the three mutational severities over the years, we found a positive correlation between the year of the hybrid lines and the count of slightly deleterious alleles in both the additive (*R* = 0.44, *P* = 5.46e−7, Wilcoxon test) and recessive (*R* = 0.27, *P* = 0.0031, Wilcoxon test) modes. However, there was no significant fluctuation in moderately and strongly deleterious mutations over breeding years (*P* > 0.01, Wilcoxon test; supplementary fig. S7, Supplementary Material online). This was more likely due to the increase in slightly deleterious mutations in parents or the increase in the frequency of slightly deleterious alleles in the hybrids over breeding years, which is consistent with a recent report documenting more common but small-effect deleterious mutations in maize landraces than in teosinte (Samayoa et al. 2021).

**Fig. 3.**
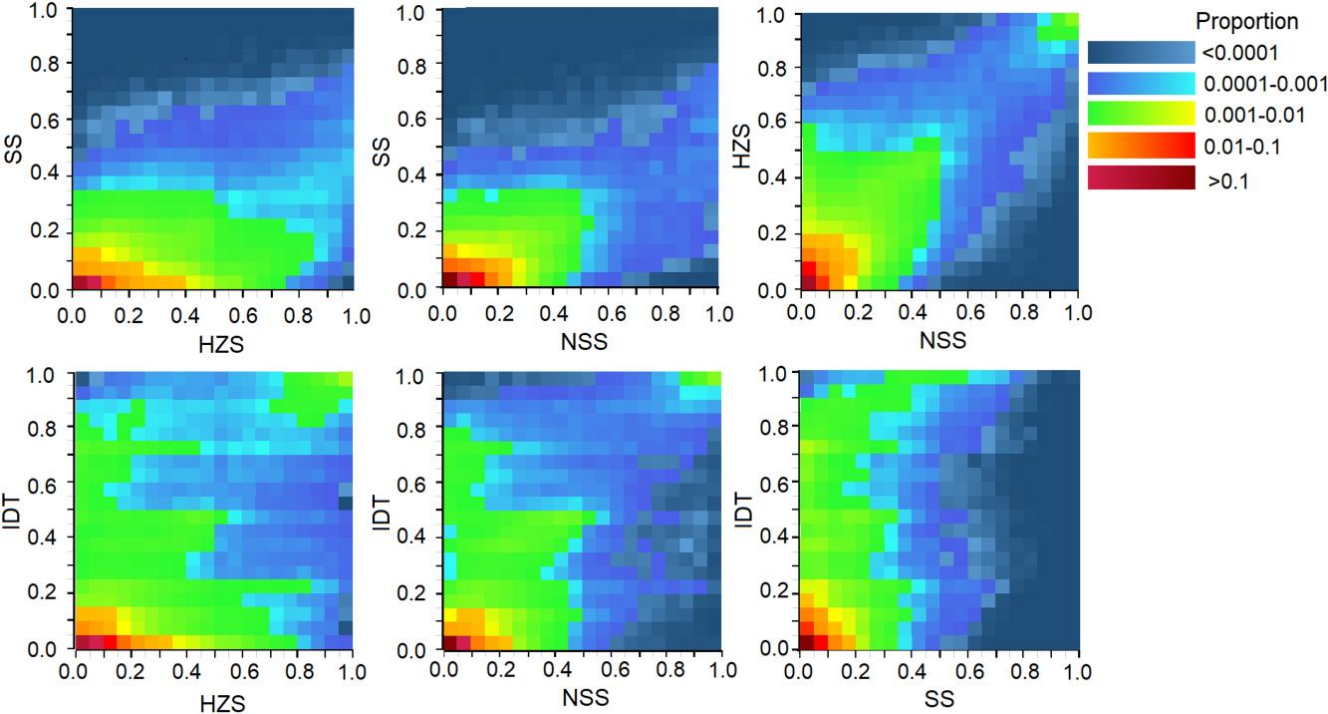
The heatmap of jSFS between pairs of heterotic groups of the ILs.

The accumulation of slightly deleterious mutations in hybrids prompted us to explore whether these mutations could be complemented when the hybrids were derived from parents belonging to distinct heterotic groups. To address this question, we first examined the joint SFS (jSFS) between pairs of heterotic groups of the ILs that were divided into four main groups: stiff stalk (SS), nonstiff stalk (NSS), iodent (IDT), and the China-specific group Huangzaosi (HZS) (Wang et al. 2020). We found asymmetric distribution of jSFS in all six pairs between heterotic groups, wherein deleterious alleles were present at low frequencies in one group but tended to be at moderate or high frequency in the other, suggesting potential complementation of slightly deleterious mutations in hybrids generated between heterotic groups (fig. 3). Conversely, symmetric distribution of jSFS was observed when the pairs were between ILs sampled from the same heterotic group (supplementary fig. S8, Supplementary Material online), indicating that complementation was more effective when hybrids were derived from parents in different heterotic groups than within a single heterotic group. Furthermore, we compared the count of deleterious mutations among the four heterotic groups and found that the SS synthetic group, represented by the most favored maize IL B73, had the lowest number of deleterious mutations among all the groups, regardless of the deleterious mutation effect under either the recessive or additive modes (supplementary fig. S9, Supplementary Material online).

### Complementation of Deleterious Mutations in Hybrids

**Fig. 4.**
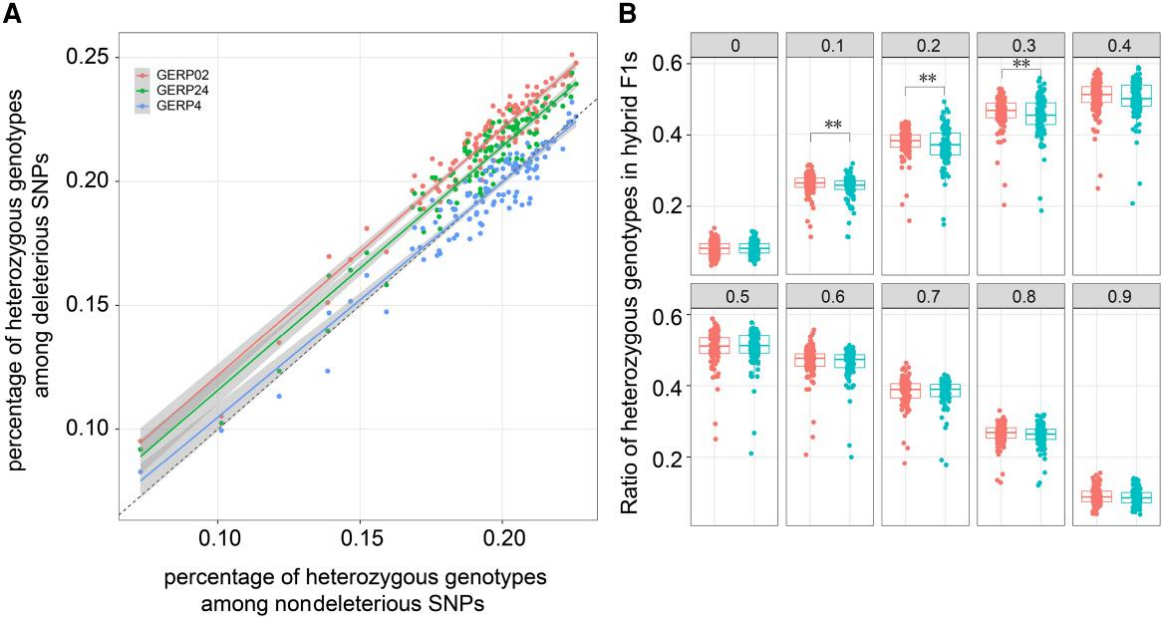
(*A*) Comparing the percentage of heterozygous genotypes of nondeleterious mutations with that of slightly, moderately, and strongly deleterious mutations in 120 hybrids. The dashed line indicates “*y* = *x*.” The other three solid lines represent the linear regression of corresponding groups of points. (*B*) Within the same DAF group, we compared the ratio of heterozygous genotypes of nondeleterious (GERP ≤ 0; indicated with the right boxes) and slightly deleterious mutations (0 < GERP ≤ 2; indicated with the left boxes).

As we observed a slight increase in deleterious mutations but an absence of reduced fitness in hybrids over breeding time, we were curious whether complementation had occurred to mask the effects of slightly deleterious mutations. To evaluate if the complementation of deleterious alleles by introducing advantageous or neutral alleles from outside populations (i.e., different heterotic pools) through hybridization explains heterosis, we compared the proportion of heterozygotes in 120 hybrids across four SNPs partitions based on the mutation effects. Notably, slightly deleterious mutations are more likely in heterozygous genotypes (measured as the percentage of slightly deleterious mutations in heterozygous genotypes vs. the total number of slightly deleterious mutations in one hybrid individual) than nondeleterious mutations, indicating a complementation effect of slightly deleterious mutations (paired *t*-test, *t* = 38, *P* < 1e−10; fig. 4*A*). Moderately deleterious mutations demonstrated more heterozygotes than nondeleterious mutations (paired *t*-test, *t* = 21, *P* < 1e−10; fig. 4*A*), but the level of differentiation decreased. Strongly deleterious mutations showed a proportion of heterozygotes comparable with that of nondeleterious mutations (paired *t*-test, *t* = 0.01, *P* = 0.98; fig. 4*A*). This reveals that complementation occurs more frequently in slightly deleterious mutations than that in strongly deleterious mutations. To account for the effect of allele frequencies on the level of heterozygosity, we divided the SNPs into ten categories based on the derived allele frequency (DAF) and evaluated whether the pattern held in each category in the 120 hybrids. Deleterious mutations with DAF between 0.1 and 0.4 showed an elevated level of heterozygotes in slightly, moderately, and strongly deleterious mutations compared with nondeleterious mutations (*P* < 0.05), but not for deleterious mutations with DAF over 0.4, suggesting that complementation occurred more frequently for lower-frequency deleterious mutations (fig. 4*B*; supplementary fig. S10, Supplementary Material online).

We analyzed the correlation of the density of deleterious mutations and heterozygous genotypes using 1-Mb nonoverlapping sliding windows normalized by SNP density across the genome. Our finding revealed that the percentage of slightly deleterious mutations explains 88% of the variation in the percentage of heterozygous genotypes in the sliding windows (*P* ≈ 0). In contrast, the correlation coefficient was only 0.27 in the case of strongly deleterious mutations (supplementary fig. S11, Supplementary Material online), indicating that the occurrence of slightly deleterious mutations was significantly correlated with the occurrence of heterozygous genotypes across the genome. This is consistent with our previous observation of an asymmetric distribution of jSFS of slightly deleterious mutations between ILs from different heterotic subgroups. In order to evaluate if the 120 hybrids were preferably chosen for better complementation, we also compared the percentage of heterozygous deleterious SNPs among all deleterious loci between the 120 actual hybrids and 120 hypothetical hybrids generated by randomly selecting two ILs as parents. The empirical hybrids exhibited a slightly higher percentage of heterozygous deleterious SNPs than the hypothetical hybrids, suggesting that the actual hybrids were selected for better complementation (supplementary fig. S12, Supplementary Material online). Our results suggest that even though the number of slightly deleterious mutations increased in hybrids over time, their fitness effects were largely alleviated by the complementation effect.

### Phenotypic Effects of Deleterious Mutations in ILs

**Fig. 5.**
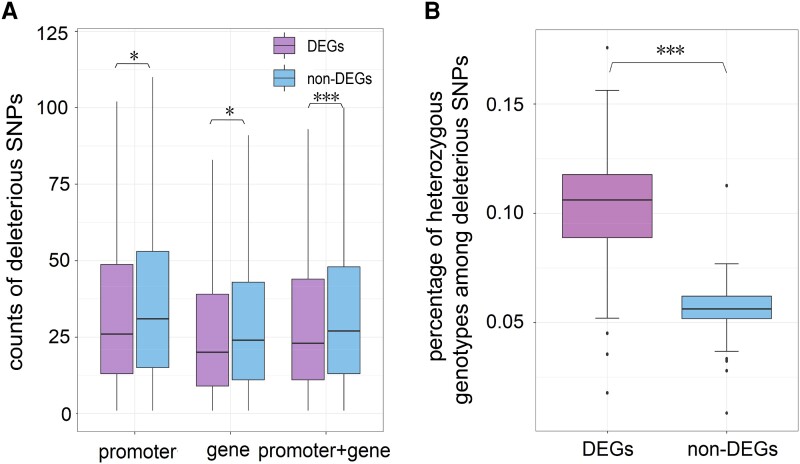
(*A*) Counts of deleterious mutations at the promoter (2-kb upstream of the gene), gene body, and combined regions for both the DEGs and non-DEGs. (*B*) Percentage of heterozygous genotypes among deleterious SNPs in both DEGs and non-DEGs genes.

To evaluate how deleterious mutations affect the traits of ILs, we assessed how the prevalence of deleterious mutations affected gene expression in the ears of elite Chinese ILs. Specifically, we examined the count of deleterious mutations in the promoter (the 2-kb upstream) and gene body regions of differentially expressed genes (DEGs) and nondifferentially expressed genes (non-DEGs) across three breeding eras (CN1 vs. CN2, CN2 vs. CN3, and CN1 vs. CN3). We found that deleterious mutations were less frequent in the promoter and gene body regions surrounding DEGs than non-DEGs (fig. 5*A*, Wilcoxon test, *P* < 0.001). We also observed no significant difference in the number of nondeleterious mutations in the region surrounding DEGs and non-DEGs, suggesting that the depletion of deleterious mutations surrounding DEGs was not caused by a reduction in overall diversity resulting from selection (supplementary fig. S13, Supplementary Material online). Furthermore, we compared the percentage of heterozygous deleterious mutations to the total number of deleterious mutations in the DEGs and non-DEGs in 120 hybrids and found that DEGs exhibited a higher percentage of heterozygous genotypes among deleterious mutations than non-DEGs (fig. 5*B*, Wilcoxon test, *P* < 0.001). In summary, our findings suggest that genes with modified expression during improvement (i.e., possible targets of selection) were less likely to accumulate deleterious mutations, and DEG genes were more likely to complement their deleterious SNPs in hybrids than non-DEG genes.

**Fig. 6.**
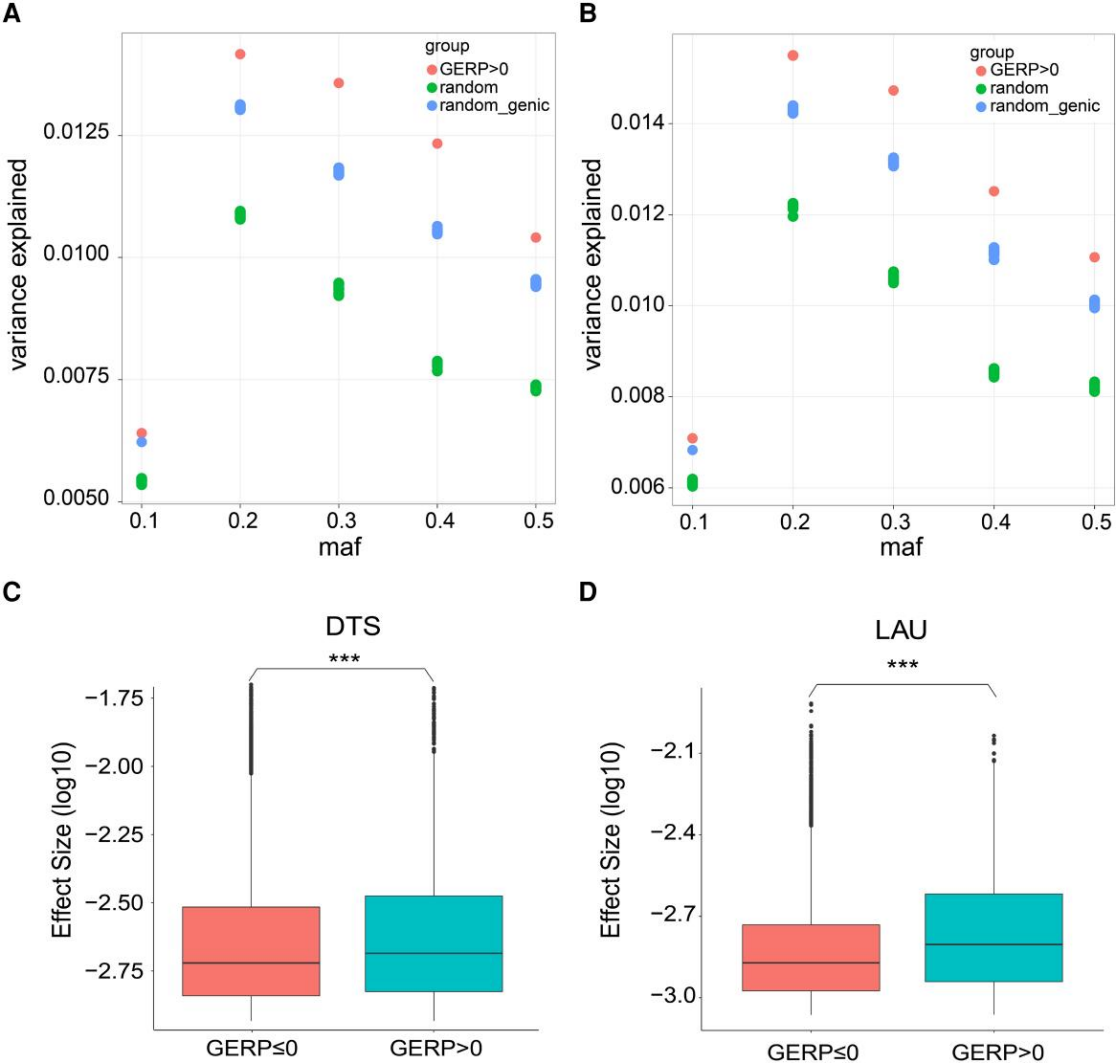
The phenotypic variance explained by deleterious mutations (GERP > 0), random SNPs across the genome, and across the genic regions in the trait DTA (*A*) and LAU (*B*). Box plots compare the effect size of deleterious (GERP > 0) and nondeleterious (GERP ≤ 0) SNPs determining the trait DTS (*C*) and LAU (*D*).

Subsequently, we investigated the direct effects of deleterious variants on agronomic phenotypes. We utilized the genomic best linear unbiased prediction (GBLUP) modeling approach (Da et al. 2014) to estimate the effect sizes and variances explained by deleterious mutations with a GERP score greater than 0 for each of the surveyed phenotypes in 350 elite ILs. We found that deleterious SNPs had larger average effects and explained more phenotypic variance than the same number of randomly selected SNPs (including SNPs with GERP scores ≤ 0) matched for allele frequencies for traits related to flowering time (days to anthesis [DTA] and days to silking [DTS]), leaf angle (LAU and lower leaf angle [LAL]), relative EH, and upper leaf number (ULN) (fig. 6; supplementary fig. S14, Supplementary Material online). However, this pattern was absent for other traits, such as total leaf number (TLN), stem diameter (SD), and tassel length (TL), and leaf length (LL), possibly because selective pressure was absent or inefficient for these traits. The difference decreased when random SNPs were selected from genic regions, better approximating the physical position of the deleterious mutations (fig. 6; supplementary fig. S14, Supplementary Material online). Our results were concordant with the enrichment of deleterious mutations in the QTL regions (*Z* test with the proportion of nondeleterious mutations in the QTL region set as *H*_0_, *Z* score = 123.27, *P* < 0.00001), which controls agronomic traits (see Materials and Methods) reported recently (Wang et al. 2020). Remarkably, the enrichment was present for all three severity levels of deleterious mutations (*P* < 0.00001). To account for the potential bias that deleterious mutations are more likely to be located in genic regions and thus overlap with QTL, we compared the counts of deleterious mutations with those of synonymous nondeleterious mutations within QTL regions and identified an enrichment of deleterious mutations in QTL regions (chi-square test, chi score = 909.59, *P* ≈ 0), which implies that deleterious mutations are more likely to affect agronomic traits in ILs.

**Fig. 7.**
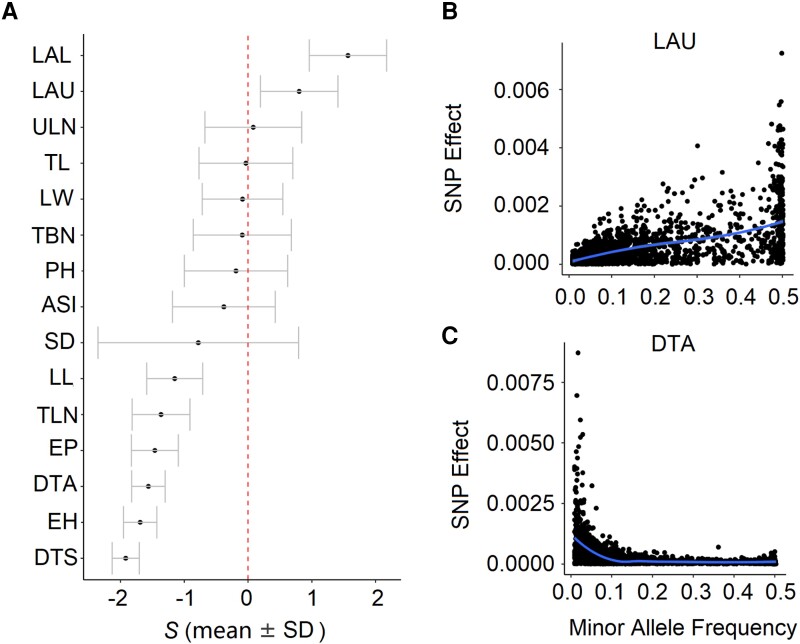
(*A*) The *S* values estimated from the GCTB analysis. Phenotypic traits with standard deviations crossed the dashed lines indicate the selective neutral traits. Points plot and loess regression (the blue line) depicting the relationship of minor allele frequency and effect size of SNPs controlling the trait LAU (*B*) and DTA (*C*).

It is largely unknown to which extent the larger effect size of deleterious mutations compared with that of neutral SNPs depends on whether traits are under selection. To answer this question, we utilized a Bayesian-based method to simultaneously estimate the joint effects of genome-wide SNPs and genetic architecture parameters for each trait in a population of 350 IL (see Materials and Methods). After pruning SNPs in high linkage disequilibrium (LD) (*r*^2^ ≥ 0.1), we used a set of independent SNPs (*n* = 500,000) for the analysis. The results indicated that two leaf angle traits (i.e., LAL and LAU) were under positive selection (*S* values > 0, i.e., effect size positively correlated with minor allele frequency) (fig. 7*A* and *B*), which was consistent with breeding efforts aimed at increasing planting density and improving leaf morphology (Duvick and Cassman 1999). Conversely, seven traits were likely to be under negative selection or stabilizing selection (*S* values < 0, i.e., effect size negatively correlated with minor allele frequency) (fig. 7*A* and *C*), including flowering time traits (DTS, DTA, and ASI). The remaining traits were likely to be neutral. Interestingly, deleterious mutations demonstrated a higher effect size than nondeleterious mutations for traits under both positive and negative selections (fig. 6*C* and *D*; supplementary fig. S15, Supplementary Material online; *P* < 1.72e−6, Bonferroni correction), suggesting that the larger effect size of deleterious mutations was independent of the direction of selection imposed on the traits.

To demonstrate how deleterious mutations may potentially impact fitness-related traits, we extracted highly deleterious SNPs with GERP > 4 within the 2-kb upstream and gene body regions of the genes located in the Genome Wide Association Analyses (GWAS) hit regions of ear weight and kernel row number (Li et al. 2022). We then compared the expression levels of the closest genes and trait values in the ILs carrying homozygous nondeleterious and deleterious alleles. This approach led to some intriguing findings where individuals carrying homozygous deleterious alleles displayed lower gene expression levels and smaller phenotypic traits than those carrying homozygous nondeleterious alleles, providing evidence of their detrimental effects on fitness-related traits. For example, *GRMZM2G316967* (*RAMOSA1 ENHANCER LOCUS2*, *REL2*), a member of the highly conserved TOPLESS (TPL) family and known to impact maize yields by regulating the development of meristem initiation and maintenance, is located within the GWAS hit region of ear weight (Liu et al. 2019; Wang et al. 2020). A highly deleterious mutation was predicted in the eighth exon of the gene (from GG to CC), resulting in a mutation in the amino acid residue from alanine to proline. ILs with CC genotypes (*n* = 21) at this locus demonstrated significantly lower ear weights than those carrying GG genotypes (*n* = 320; Wilcoxon test; *P* = 0.026) and lower gene expression of *GRMZM2G316967* (Wilcoxon test; *P* = 0.039) (supplementary fig. S16, Supplementary Material online). Similarly, *GRMZM2G019874*, which encodes a pentatricopeptide repeat protein essential for seed endosperm development and plant growth in maize, is located within the GWAS hit region of the kernel row number (Gutiérrez-Marcos et al. 2007; Wang et al. 2020). A highly deleterious mutation was predicted at the promoter region (from CC to GG). ILs with CC genotypes (*n* = 21) at this locus demonstrated significantly fewer kernel rows than those carrying GG genotypes (*n* = 326; Wilcoxon test; *P* = 0.034) and lower gene expression of *GRMZM2G019874* (Wilcoxon test; *P* = 0.023) (supplementary fig. S16, Supplementary Material online). Moreover, *GRMZM5G877077*, a *P*-loop containing nucleoside triphosphate hydrolase superfamily protein highly expressed in tassels and anthers, is a candidate gene for kernel row number in maize (An et al. 2020). A highly deleterious mutation was predicted in the third intron of *GRMZM5G877077* (from TT to CC). ILs with CC genotypes (*n* = 39) at this locus demonstrated significantly smaller kernel row numbers and lower gene expression (Wilcoxon test; *P* = 0.008) than those carrying TT genotypes (*n* = 303; *P* = 0.032; Wilcoxon test; supplementary fig. S16, Supplementary Material online).

## Discussion

In modern maize breeding, inbreeding and hybridization are widely used to maintain desired traits and enhance hybrid performance. However, inbreeding can lead to inbreeding depression due to the exposure of recessive deleterious alleles, unless negative selection removes these alleles. Hybridization can diminish inbreeding depression by complementing deleterious alleles, which reduces their impact. This study investigated the genomic consequences of inbreeding and hybridization in maize ILs across decades of improvement.

Intriguingly, the dynamic trend of deleterious mutations across breeding eras depends on their mutational effects. Slightly deleterious mutations were accumulated in both ILs and individual hybrids and were complemented by neutral or favorable alleles when their frequencies were low in the hybrids. In contrast, strongly deleterious mutations were purged by negative selection as inbreeding exposed recessive deleterious mutations to negative selection in ILs. The asymmetric distribution of jSFS between distinct heterotic groups of maize ILs and their high occurrence in heterozygous genotypes in hybrids supported the complementation of slightly deleterious mutations, which is a contributing factor to the continuous improvement in key agronomic traits in commercial varieties. Our study also demonstrated that genes with modified expression across breeding eras were depleted of deleterious mutations, suggesting that functionally important genes experience stronger evolutionary constraint. Deleterious mutations were found to have a greater phenotypic effect than genomic/genic neutral SNPs, and the larger effect size of deleterious mutations was independent of the directionality of selection imposed on different traits. In summary, this study provides a detailed map of the fluctuation of deleterious mutations along the time dimensions of modern breeding, illustrating the complementation of slightly deleterious mutations as an explanation for heterosis at the genomic scale and assessing the effect of deleterious mutations on gene expression and phenotypic traits.

The empirical evidence for the effectiveness of purging deleterious mutations is limited and mainly restricted to rare and endangered species, as reported by previous studies (Hedrick and Garcia-Dorado 2016; Grossen et al. 2020). For instance, island foxes have successfully purged highly deleterious mutations despite recent bottlenecks. However, failure to purge strongly deleterious mutations in fragmented populations may lead to detrimental consequences (Robinson et al. 2019). In the case of maize ILs, we observed a gradual decline in effective population size and genetic diversity across breeding eras (Wang et al. 2020), which overall reduced the burden of highly deleterious mutations and elevated the burden of weakly deleterious mutations, consistent with some recent reports (Roessler et al. 2019). The effectiveness of purging deleterious mutations depends on the selection coefficient magnitude and effective population size (*N_e_* ∗ *s*). Strongly deleterious mutations with a large selection of coefficients were quickly purged by negative selection, while weakly deleterious mutations with a smaller selection coefficient were “nearly neutral” when *N_e_* ∗ *s* < 1, and their behavior was dependent on the effective population size (Lohmueller 2014; Huber et al. 2020). Although purging highly deleterious mutations can prevent short-term fitness reduction, the accumulation of slightly deleterious mutations may lead to the long-term effects of inbreeding depression.

Heterosis is the opposite of inbreeding depression. The effects of inbreeding depression can be alleviated when a hybrid is formed, demonstrating superior characteristics (Springer and Stupar 2007). Here, we demonstrated that weakly deleterious mutations at low frequencies are more likely to be complemented in hybrids, possibly due to the dominance effect, which is a plausible explanation for heterotic phenotypes. Our results are consistent with previous reports showing that dominance is pervasive in maize (Yang et al. 2017; Ramstein et al. 2020; Zhu et al. 2022), but we are more cautious when considering both mutational severity and allele frequencies. Epistasis cannot be excluded as a mechanism contributing to heterosis. A recent study implied that deleterious alleles in one gene could suppress the expression of another gene, whereas in hybrids (heterozygous status of the deleterious allele), the suppression was alleviated, and the expression of the interaction gene was recovered (Xiao et al. 2021). Therefore, the heterosis in maize is possibly a compound effect of multiple genetic mechanisms. Further investigation of the effects of structural variance (i.e., gene presence/absence variation) and gene expression complementation on heterotic phenotypes will facilitate our understanding of the genetic basis of heterosis.

There is a gap between studies on quantitative genetics and genetic load based on genomic sequences, especially in nonmodel species, such as trees (Liu et al. 2022). The former focuses on direct fitness effects and is often limited to a few crop species, while the latter centers on comparative data sets of species or populations but lacks direct fitness estimates. Efforts have been made to address this gap in maize (Yang et al. 2017) and sorghum (Valluru et al. 2019). Encompassing putatively deleterious mutations in genomic selection models can improve the prediction accuracy of fitness-related traits (Yang et al. 2017; Wu et al. 2023), and the contribution of deleterious mutations to phenotypic variance depends on the genetic architecture of the traits (Valluru et al. 2019). Our work adds to the cumulative evidence of the stronger effects of deleterious mutations on phenotypic diversity than random SNPs (Yang et al. 2017; Zhang et al. 2019; Wu et al. 2023). Although functional studies of specific deleterious mutations provide direct evidence of their fitness consequences (Zhang et al. 2019), it is laborious to experimentally validate pyramids of deleterious mutations (Bertorelle et al. 2022). In this study, we have provided several candidate deleterious mutations for further experimental validation by thoroughly examining strongly deleterious mutations in GWAS hit regions of yield-related traits and their impact on gene expression and the trait itself.

The trend of deleterious mutations in maize is likely influenced by the species’ reproductive mode and effective population size. Recent studies (Kim et al. 2020; Lozano et al. 2021; Sun et al. 2023) indicate that the primarily selfing species such as sorghum and soybean have experienced a reduction in deleterious mutations postdomestication and improvement, contrary to the “domestication cost” theory. The inbreeding mode of these species exposes recessive deleterious mutations to purifying selection, leading to a decrease in their frequencies. The effective population size also plays a significant role in determining the prevalence of deleterious mutations, with larger populations able to purge them more effectively. Small effective population sizes, as seen in endangered or bottlenecked populations, can fail to purge deleterious mutations and cause the population to be on the brink of extinction (Robinson et al. 2018). Therefore, reproductive mode and effective population size should be considered when transferring our conclusions to other crops or species.

Our findings can be leveraged to systematically detect and remove/repair deleterious mutations in maize breeding using high-throughput genomic editing or conventional breeding involving parental lines with fewer deleterious mutations. However, prioritizing deleterious mutations for removal remains a challenge, as there are thousands of deleterious SNPs to consider. Based on our results, mutations with large effects, medium-to-high frequency in the population, and effects on gene expression and phenotypic traits should be given high priority in modern breeding programs. Overall, this study sheds light on the importance of repairing unseen deleterious mutations in future breeding efforts to prevent long-term fitness decay (Wallace et al. 2018).

## Materials and Methods

### Sample, Phenotypic, and Genotypic Data

The whole-genome resequencing and 15 phenotypic data, including ASI, DTA, DTS, EH, EP, LAL, LAU, LL, leaf width (LW), PH, TBN, TL, SD, TLN, and ULN (supplementary table S3, Supplementary Material online), for 350 public elite maize ILs were previously published (Wang et al. 2020). These 350 ILs were divided into six classes based on their historical information, with 30 ILs in the CN_1960&70s class, 95 ILs in the CN_1980&90s class, 53 ILs in the CN_2000&10s class, 74 ILs in the Public_US class, 89 ILs in the Ex-PVP class, and nine ILs with unknown historical information. RNA-seq data were generated for the ears of the Chinese elite ILs, which were grown in Langfang, Hebei Province, China. Unpollinated ears at the silking stage were collected from each sample with three biological replicates, frozen in liquid nitrogen, and stored at −80 °C before RNA extraction. Total RNA was extracted using the TRIzol reagent (Life Technologies), and RNA-seq libraries were constructed using manufacturer-specified methods from Illumina and sequenced using the HiSeq 2000 platform to generate 150-nucleotide paired-end reads.

Additionally, subsets of the 350 ILs were selected for crossing, which resulted in the generation of 120 hybrids (supplementary table S1, Supplementary Material online) that were historically popular commercial varieties planted in China. To evaluate the performance of these hybrids, we measured 12 phenotypic traits in the plants grown at Langfang, Hebei Province, China (the same location as the ILs) in 2017. These traits included DTA, DTS, ASI, LAL, LAU, LW, LL, PH, EH, EP, TBN, and TL. The genotypes of the 120 hybrids were inferred from the genotypes of their parents based on their known kinship relationships.

### Inbreeding Coefficients and ROH

The inbreeding coefficient was calculated using VCFtools (Danecek et al. 2011). SNPs were downsampled to include one SNP in a 2-kb window to identify segments representing homozygosity by descent (i.e., autozygosity) rather than by chance. SNPs in Variant Call Format were converted into Plink format using VCFtools (Danecek et al. 2011). PLINK (http://pngu.mgh.harvard.edu/purcell/plink/) (Purcell et al. 2007) was used to identify segments of ROH in a window containing 20 SNPs, among which the maximum number of missing SNPs was set to 2 and the maximum number of heterozygous sites was set to 1. The shortest length of the final ROHs was set to be 300 kb.

### Prevalence of Deleterious Mutations

In order to characterize the number of deleterious mutations across stratified breeding eras and among subgroups based on population structure analyses, we first quantified the deleterious mutations using the approach of GERP (Cooper et al. 2005). In brief, we aligned 13 plant genomes including *Z. mays, Coelorachis tuberculosa, Vossia cuspidata, S. bicolor, Oryza sativa, Setaria italica, Brachypodium distachyon, Hordeum vulgare, Musa acuminata, Populus trichocarpa, Vitis vinifera, Arabidopsis thaliana*, and *Panicum virgatum* to the maize reference genome. First, each nonmaize sequence was aligned to the maize reference genome using BWA mem, followed by reordering and reorientation of the nonmaize sequences to ensure optimal alignment. Any nonmaize sequences that failed to exhibit detectable similarity to any region of the maize genome were eliminated. Then, a global multiple alignment of the rearranged sequences was performed using MAFFT (Katoh et al. 2002), and the maximum likelihood branch lengths were obtained with RAxML (Stamatakis 2014), using the HKY 85 model of nucleotide substitution, in accordance with the species topology tree. The resulting neutral rate was calculated as an estimate of the total number of neutral substitutions per site captured by the tree. Finally, resistance scores were computed based on the conservation level of nucleotides of 12 species aligned to the *Z. mays* genome with the reference genome ejected from the calculation. Deleterious mutations were divided into three categories: 1) SNPs with GERP between 0 and 2 were treated as slightly deleterious mutations; 2) SNPs with GERP between 2 and 4 as moderately deleterious mutations; and 3) SNPs with GERP greater than 4 as strongly deleterious mutations. We counted the number of deleterious mutations in two modes: the additive mode with deleterious alleles in both homozygous and heterozygous genotypes and the recessive mode with deleterious alleles only in homozygous genotypes. For each GERP partition, we calculated the mutation load of ILs across three breeding eras in China (1960s and 1970s; 1980s and 1990s; 2000s and 2010s), two breeding eras in the United States (Public US and extended PVP) and of 120 hybrids in both additive and recessive modes. The same procedures were applied to enumerate the mutation load among four population subgroups (HZS, IDT, NSS, and SS) based on previous population structure analyses (Wang et al. 2020).

Sorting Intolerant From Tolerant 4G (SIFT4G) was also employed to annotate SNPs, which were located in protein-coding regions (Vaser et al. 2016). Uniref90 was used as a reference protein database. SIFT4G utilizes protein alignments to identify conserved amino acids and provides a score ranging from 0 to 1 for each SNP. The annotation file of B73 APGv3 in gff3 format was downloaded from Ensembl Plants (https://plants.ensembl.org) and converted to gtf format with gffread (Pertea and Pertea 2020). Nonsynonymous mutations with a SIFT score < 0.05 were assigned as deleterious SNPs. The genome of *S. bicolor* was used as the ancestral allele state to polarize deleterious SNPs, and the derived alleles with SIFT score < 0.05 were considered as “deleterious.”

To compare the burden of fixed deleterious variants among different breeding eras, we randomly selected 30 accessions from the CN2 and CN3 populations to match the sample size of CN1. Similarly, we selected 74 accessions from the population of Ex-PVP to match the sample size of Public_US. Then, we detected the fixed deleterious SNPs with allele frequencies of 1 using PLINK (Purcell et al. 2007). Finally, we calculated and compared the number of fixed deleterious SNPs in each era population.

### The Frequency of Deleterious Mutations

The comparison of deleterious mutations across breeding eras and SNP groups was not only limited to counting the number of mutations but also involved evaluating the DAF of these mutations. Specifically, DAF was calculated across three breeding eras in China (CN1 [Chinese ILs developed in 1960s and 1970s], CN2 [1980s and 1990s], and CN3 [2000s and 2010s]) and two breeding eras in the United States (Public_US and Ex-PVP) and among SNP groups with different mutation effects (nondeleterious SNPs, slightly deleterious alleles [0 < GERP ≤ 2], moderately deleterious alleles [2 < GERP ≤ 4], and strongly deleterious alleles [GERP > 4]). To reduce sample size bias, we downsampled the Chinese breeding eras to 30 individuals (the smallest size in CN1) and the US breeding eras to 74 individuals. The DAF was calculated using the program SoFoS (https://github.com/reedacartwright/SoFoS), which utilizes the genomes of *S. bicolor* for SNP polarization. Finally, the DAF was divided into 20 equal bins for density calculation.

To detect potential complementation effects in hybrids when the parents were from different heterotic groups, we examined the jSFS of SS, NSS, IDT, and the Chinese-specific group HZS using easySFS (https://github.com/isaacovercast/easySFS). To remove the bias of population size on the DAF, we downsampled to the smaller sample size between the paired groups.

### Phenotypic Effect Size

The additive effect (*a*) and dominant effect (*d*) of deleterious and nondeleterious mutations were estimated for 15 traits in 350 ILs and 12 traits in 120 hybrids using a GBLUP model implemented in the software GVCBLUP (Wang et al. 2014) with default parameters. We filtered for SNPs whose additive effect was nonzero and whose explained phenotypic variance was bigger than the mean explained variance per SNP across the whole genome. Since this approach could generate a big variation in the degree of dominance (*k*) at both the trait and the SNP level, we filtered for SNPs with |*k* = *d*/*a*| < 2. To compare the total variance explained by deleterious SNPs (GERP > 0) and that by nondeleterious SNPs (GERP ≤ 0), we randomly sampled ten data sets of equal-sized nondeleterious SNPs with the same allele frequency (in bins of 10). For each randomly selected data set, we repeated the above calculation of SNP effects and explained variance in GVCBLUP. Additionally, to better approximate the genomic location of deleterious mutations, we randomly sampled neutral SNPs from the genic regions using the same approach as those from the whole-genomic background.

### Comparison of Deleterious Mutation between DEGs and Non-DEGs

Total RNA of ears of 131 maize inbreed lines was extracted and RNA-seq was performed on Illumina HiSeq 2000 platform. Paired-end RNA-seq reads were processed using Trimmomatic software (Bolger et al. 2014) to remove adapter sequences and low-quality bases, followed by mapping to the B73 AGP v4 reference genome (Jiao et al. 2017) using Hisat2-2.0.4 (Kim et al. 2015) with default parameters. The BAM files of uniquely mapped reads were used to generate read counts with HTSeq (Anders et al. 2015). Gene expression levels were measured in Fragments Per Kilobase of transcript per Million mapped reads values using the DESeq2 Bioconductor package (Love et al. 2014). DEGs were identified using DESeq2 (Love et al. 2014) with a significance threshold of an adjusted *P* value of 0.05. The tool of Liftoff (Shumate and Salzberg 2021) was used to convert coordinates of genes based on B73 APG v4 into that of B73 APG v3. The count of deleterious mutations was obtained by intersecting the coordinates of genes and their 2-kb upstream regions with the coordinates of the deleterious mutations using bedtools (Quinlan 2014). The counts of deleterious mutations in the promoter region (2-kb upstream of the gene), gene body region, and the combined two regions were compared between DEGs and non-DEGs. The percentage of heterozygous genotypes among deleterious SNPs was estimated for both DEGs and non-DEGs.

### Estimation of the Genetic Architecture Parameters of the Phenotypic Traits

A Bayesian linear mixed model was used to estimate several parameters related to SNP-based heritability, polygenicity (proportion of SNPs with nonzero effects) and the relationship between SNP effect size and minor allele frequency, using the GCTB software (Zeng et al. 2018). The analysis was conducted using phenotypic and genotypic data from 350 elite maize ILs from China and the United States, representing multiple eras of germplasm (Wang et al. 2020). Prior to the analysis, SNPs in high LD were pruned using the “indep-pairwise” command of the PLINK software (Purcell et al. 2007) with an LD threshold of *r^2^* = 0.1. We conducted the GCTB analysis by incorporating the first three principal components (PCs) that were calculated using genome-wide markers to control for population structure. The “BayesS” model was then used in the GCTB analysis, with a chain length of 410,000 and burn-in of 10,000.

## Supplementary Material

msad170_Supplementary_Data

## Data Availability

The RNA-seq raw reads have been deposited in NCBI SRA (PRJNA783356). ***Conflict of interest statement.*** The authors declare that the research was conducted in the absence of any commercial or financial relationships that could be construed as a potential conflict of interest.
