# Supplementary material for: Unraveling Prevalence and Effects of Deleterious Mutations in Maize Elite Lines across Decades of Modern Breeding
Source: Mol Biol Evol. 2023 Jul 26;40(8):msad170. doi: 10.1093/molbev/msad170 (PMC10414807; doi:10.1093/molbev/msad170)
Supplement: msad170_Supplementary_Data [file msad170_supplementary_data.zip › supplementary.pdf]

Supplementary table legends

Table S1. The pedigree information and phenotypes of 120 hybrides.

Table S2. Comparison of the mean values and the percentage of reduction of four traits between hybrids and mid-parents across breeding eras.

Table S3. The abbreviations and full description of surveyed phenotypes.

Table S2

traits	group	CN1960&70s	CN2000&10s	Percentage of reduction (%)
ASI	hybrids	2.226	1.170	47.4
	mid-parents	1.927	1.289	33.1
EP	hybrids	49.505	43.674	11.8
	mid-parents	39.755	35.305	11.2
LAU	hybrids	31.979	20.000	37.5
	mid-parents	32.629	22.630	30.6
TBN	hybrids	20.479	12.083	41.0
	mid-parents	14.356	8.923	37.8

Table S3

Abbreviation	Traits	Description
ASI	Anthesis to silking interval	Days interval of anthesis to silking for a plot
DTA	Days to anthesis	Days to half of plants anthesis in a plot
DTS	Days to silking	Days to half of plants silking in a plot
EH	Ear height	Height of ear setting node to earth surface
EP	Relative ear height	EH/PH(%)
LAL	Lower leaf angle	Angle between midrib and lower stem of 1st leaf above ear node
LAU	Upper leaf angle	Angle between midrib and upper stem of 1st leaf above ear node
LL	Leaf length	Leaf length of 1st leaf above ear node
LW	Leaf width	Leaf width of 1st leaf above ear node
PH	Plant height	Height of tassel tip to earth surface
SD	Stem diameter	Diameter of 4th visible stem upon earth surface
TBN	Tassel branch number	The primary branch number of tassel
TL	Tassel length	Length between tassel tip to first tassel branch node
TLN	Total leaf number	Total leaf number of a plant
ULN	Upper leaf number	Number of leaves above upper most ear

Supplementary figure legends

FIG. S1. Linear regression of trait values between hybrids and their mid-parents. ASI: anthesis to silking interval; EP: relative ear height; LAU: upper leaf angle; TBN: tassel branch number.

FIG. S2. Count of both the homozygous and heterozygous deleterious alleles in ILs from the stratified eras in China and the US. Each box represents the median and interquartile range (IQR). The whiskers represent the range of 1.5 times IQR and each dot represents a sample.

FIG. S3. The inbreeding coefficient (A) and sum of runs of homozygosity (ROH) across the breeding eras in both China and the US. Each box represents the median and interquartile range (IQR). The whiskers represent the range of 1.5 times IQR and each dot represents a sample.

FIG. S4. Count of all and strongly deleterious alleles in ILs from the stratified eras in China and the US. CN_1960&70s, CN_1980&90s, and CN_2000&10s represent Chinese maize inbred lines collected from three breeding periods in China: the 1960s and 1970s, the 1980s and 1990s, and the 2000s and 2010s, respectively; Public_US and Ex-PVP represent US maize inbred lines collected during two breeding periods: before 2003 and after 2003. The centre line indicates the median, the box limits indicate the upper and lower quartiles, and the whiskers indicate 1.5 times the interquartile range.

FIG. S5. The fixed load in both China and United States maize inbred lines. CN_1960&70s, CN_1980&90s, and CN_2000&10s represent Chinese maize inbred lines collected from these years in China: the 1960s and 1970s, the 1980s and 1990s, and the 2000s and 2010s, respectively; Public_US and Ex_PVP represent US maize inbred lines collected during two breeding periods: before 2003 and after 2003. The centre line indicates the median, the box limits indicate the upper and lower quartiles, and the whiskers indicate 1.5 times the interquartile range.

FIG. S6. The derived allele frequency of four types of mutations in maize. GERP02 represents slightly deleterious alleles; GERP24 represents moderately deleterious alleles; GERP4 represents strongly deleterious alleles; nonDeleterious represents non-deleterious alleles.

FIG. S7. Linear regression of the counts of deleterious mutations over breeding years in 120 hybrids under both the additive (A) and recessive (B) modes. Each point represents a sample. The solid lines and gray area define the best-fit regression line and its 95% confidence interval, respectively.

FIG. S8. The heatmap of jSFS between two randomly sampled groups of ILs from the same heterotic group - Stiff Stalk.

FIG. S9. Count of all (A) and homozygous (B) deleterious alleles in five sub-groups based on population structure analyses. SS: Stiff Stalk; NSS: Non-Stiff Stalk; IDT: Iodent; HZS: Huangzaosi; Mix: unknown group.

FIG. S10. Comparison of the ratio of heterozygous genotypes in 120 hybrids between the moderately and non-deleterious mutations (A) as well as between the strongly and non-deleterious mutations (B).

FIG. S11. Percentage of slightly (A) and strongly (B) deleterious SNPs vs. percentage of heterozygous SNPs in 1Mb non-overlapping sliding windows in 120 hybrids. Each point represents a 1 Mb window. GERP02 represents slightly deleterious alleles; GERP4 represents strongly deleterious alleles.

FIG. S12. Percentage of heterozygous deleterious SNPs among all deleterious SNPs in both the 120 empirical and 120 hypothetical hybrids. Each point represents a sample. Each box represents the median and interquartile range (IQR). The whiskers represent the range of 1.5 times IQR and the dots beyond the whiskers are outliers.

FIG. S13. Counts of non-deleterious mutations at the promoter (2kb upstream of the gene), gene body and combined regions for both the differentially expressed (DEGs) and non-differentially expressed (non-DEGs) genes. Significant levels are indicated above the boxes and "ns" indicates $p > 0.05$.

FIG. S14. Comparison of SNP effect size contributing to all surveyed traits among four SNP categories: non-deleterious (GERP0), slightly (GERP02), moderately (GERP24) and strongly (GERP4) deleterious mutations. For the abbreviations of traits, please refer to Table S3.

FIG. S15. The phenotypic variance explained by deleterious mutations, random SNPs across the genome and across the genic regions in the trait DTS (A), LAL(B), EP (C) and ULN (D). maf: minor allele frequency; DTS: days to silking; LAL: lower leaf angle; EP: relative ear height; ULN: upper leaf number.

FIG. S16. The phenotypic effects of three strongly deleterious variants (GERP > 4; the physical positions were marked with the grey boxes in the plot subtitle) in 350 ILs. **A.** The expressional

level of the closest genes linked with the strongly deleterious mutations. **B.** The BLUP values of ear weight and kernel row number of inbred lines with the corresponding homozygous non-deleterious (homo non-del; blue boxes) and homozygous deleterious (homo del; red boxes) alleles. The closest genes were indicated at the top of each column. BLUP: Best Linear Unbiased Prediction; FPKM: Fragments Per Kilobase of exon model per Million mapped fragments.

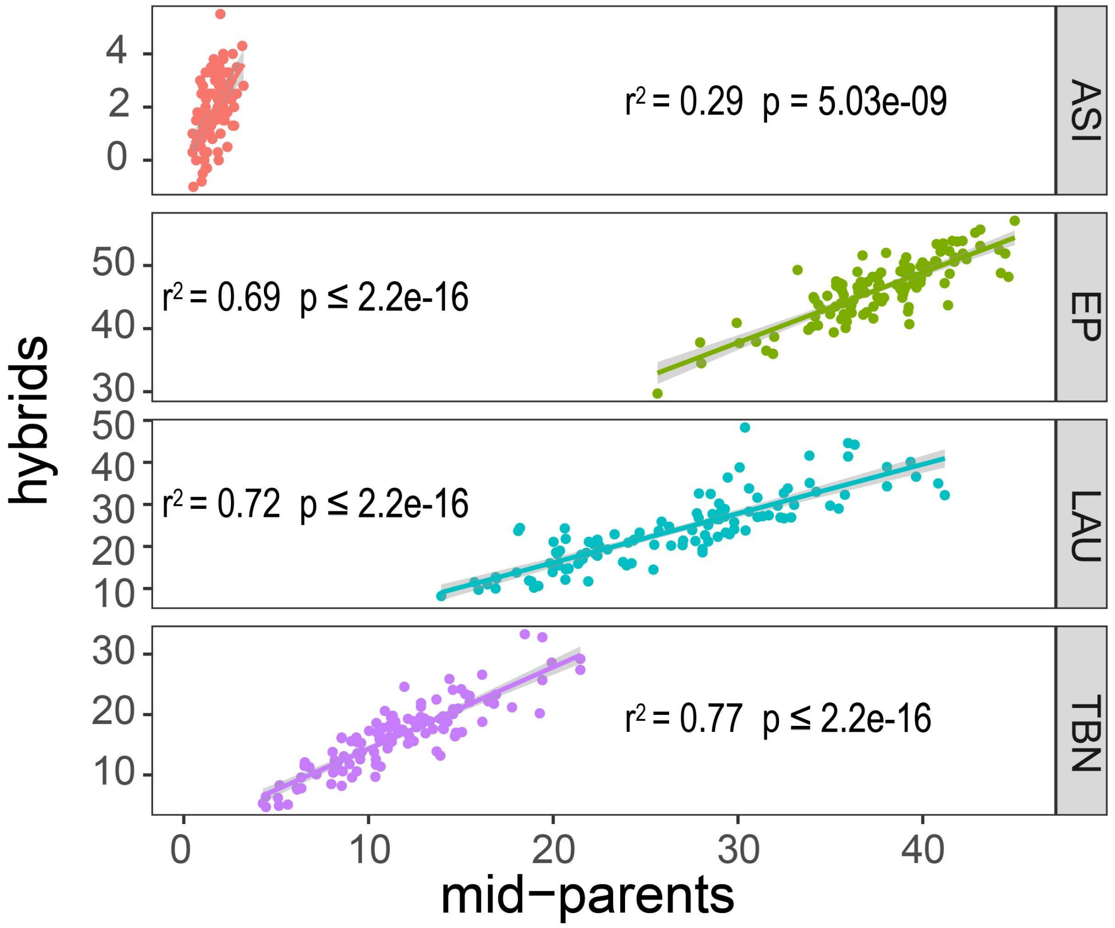
r2 = 0.29 p = 5.03e-09
ASI
r2 = 0.69 p ≤ 2.2e-16
EP
r2 = 0.72 p ≤ 2.2e-16
LAU
r2 = 0.77 p ≤ 2.2e-16
TBN
hybrids
mid−parents

FIG.S1

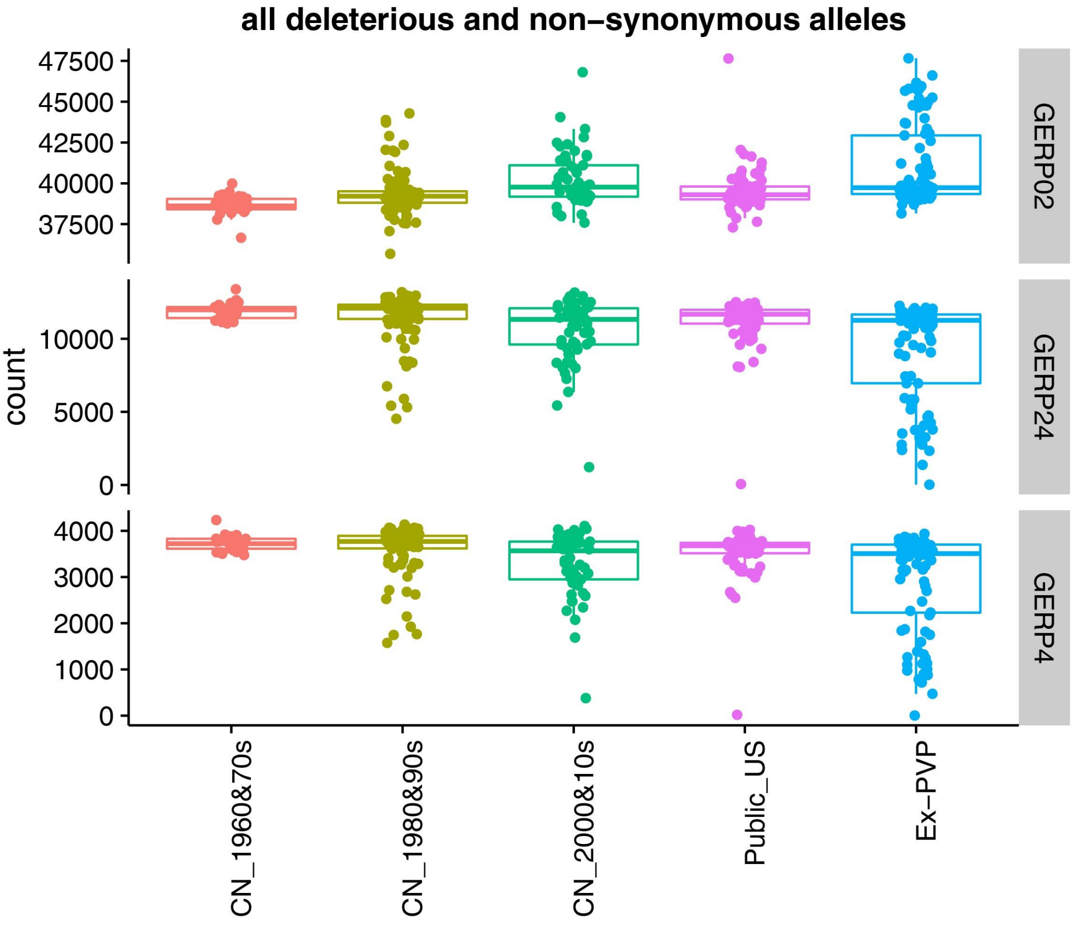
all deleterious and non-synonymous alleles
count
47500
45000
42500
40000
37500
10000
5000
0
4000
3000
2000
1000
0
GERP02
GERP24
GERP4
CN_1960&70s
CN_1980&90s
CN_2000&10s
Public_US
Ex-PVP

FIG.S2

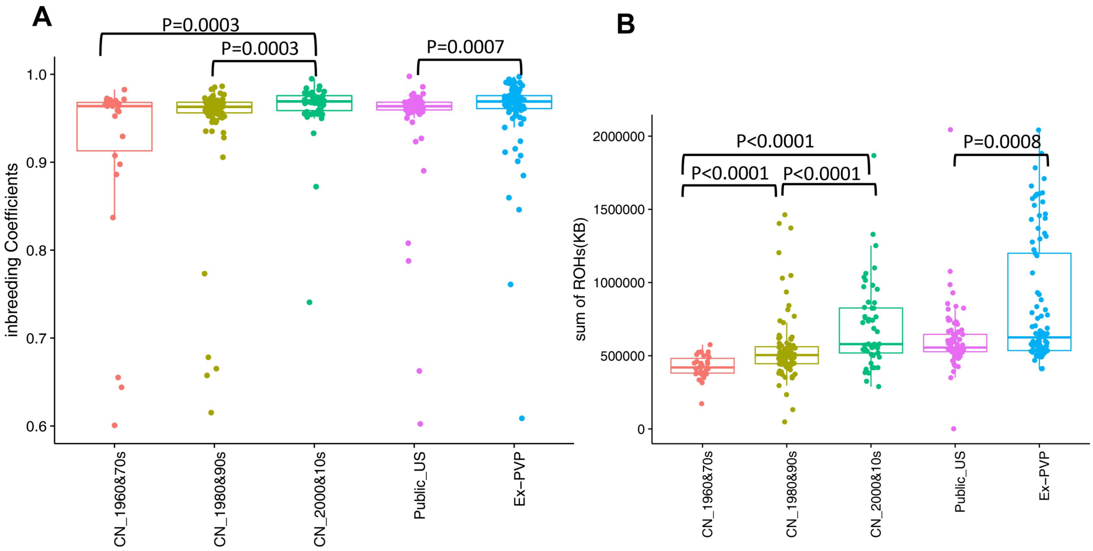
A
P=0.0003
P=0.0003
P=0.0007
inbreeding Coefficients
1.0
0.9
0.8
0.7
0.6
CN_1960&70s
CN_1980&90s
CN_2000&10s
Public_US
Ex−PVP
B
P<0.0001
P<0.0001
P<0.0001
P=0.0008
sum of ROHs(KB)
2000000
1500000
1000000
500000
0
CN_1960&70s
CN_1980&90s
CN_2000&10s
Public_US
Ex−PVP

FIG.S3

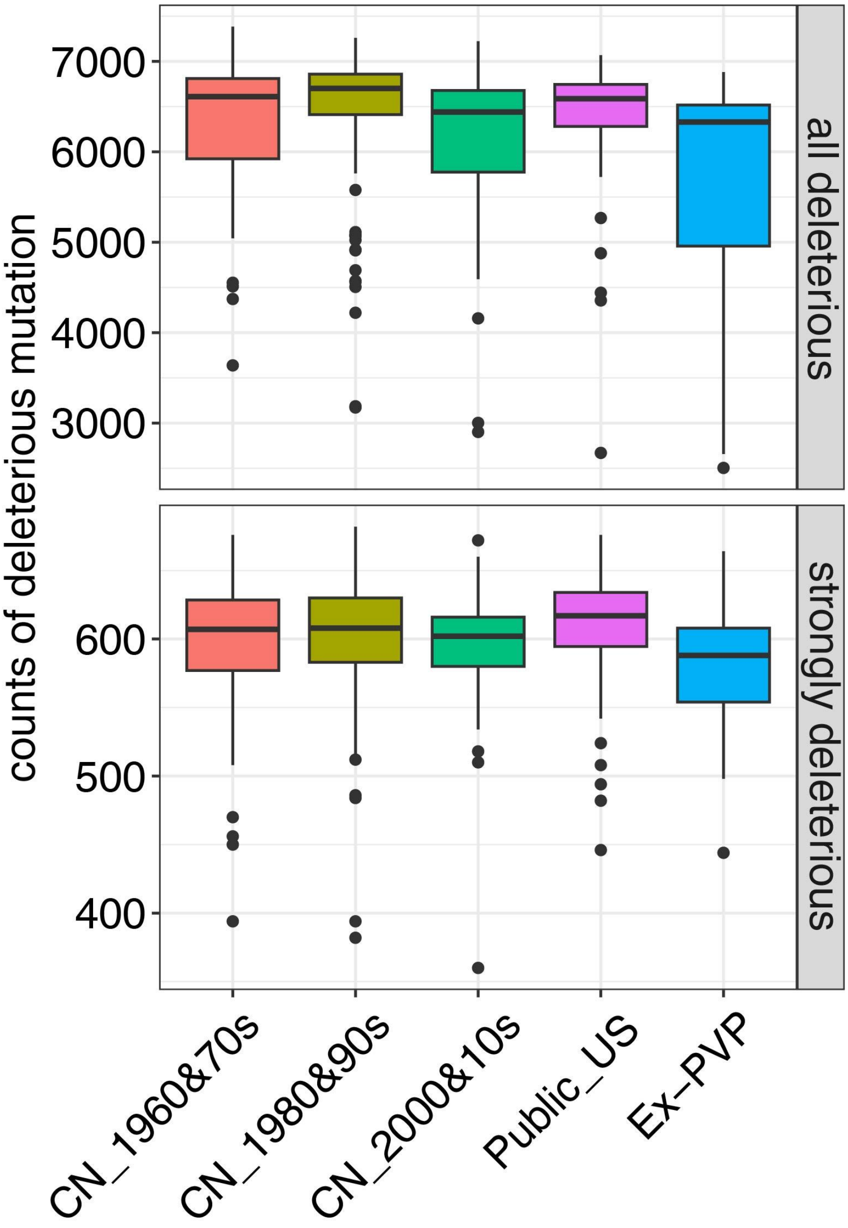

counts of deleterious mutation
7000
6000
5000
4000
3000
600
500
400
all deleterious
strongly deleterious
CN_1960&70s
CN_1980&90s
CN_2000&10s
Public_US
Ex–PVP

FIG.S4

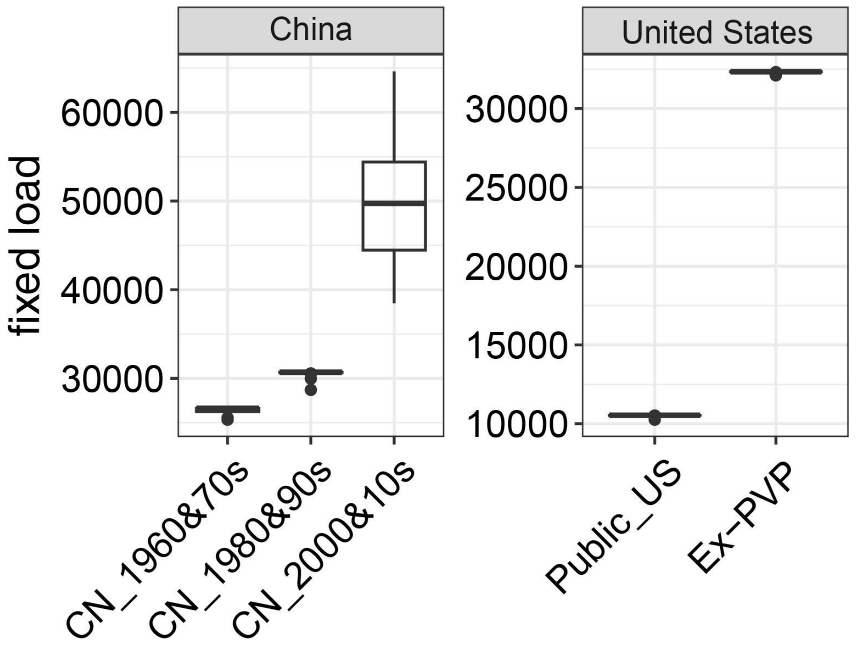
China
United States
fixed load
60000
50000
40000
30000
30000
25000
20000
15000
10000
CN_1960&70s
CN_1980&90s
CN_2000&10s
Public_US
Ex–PVP

FIG.S5

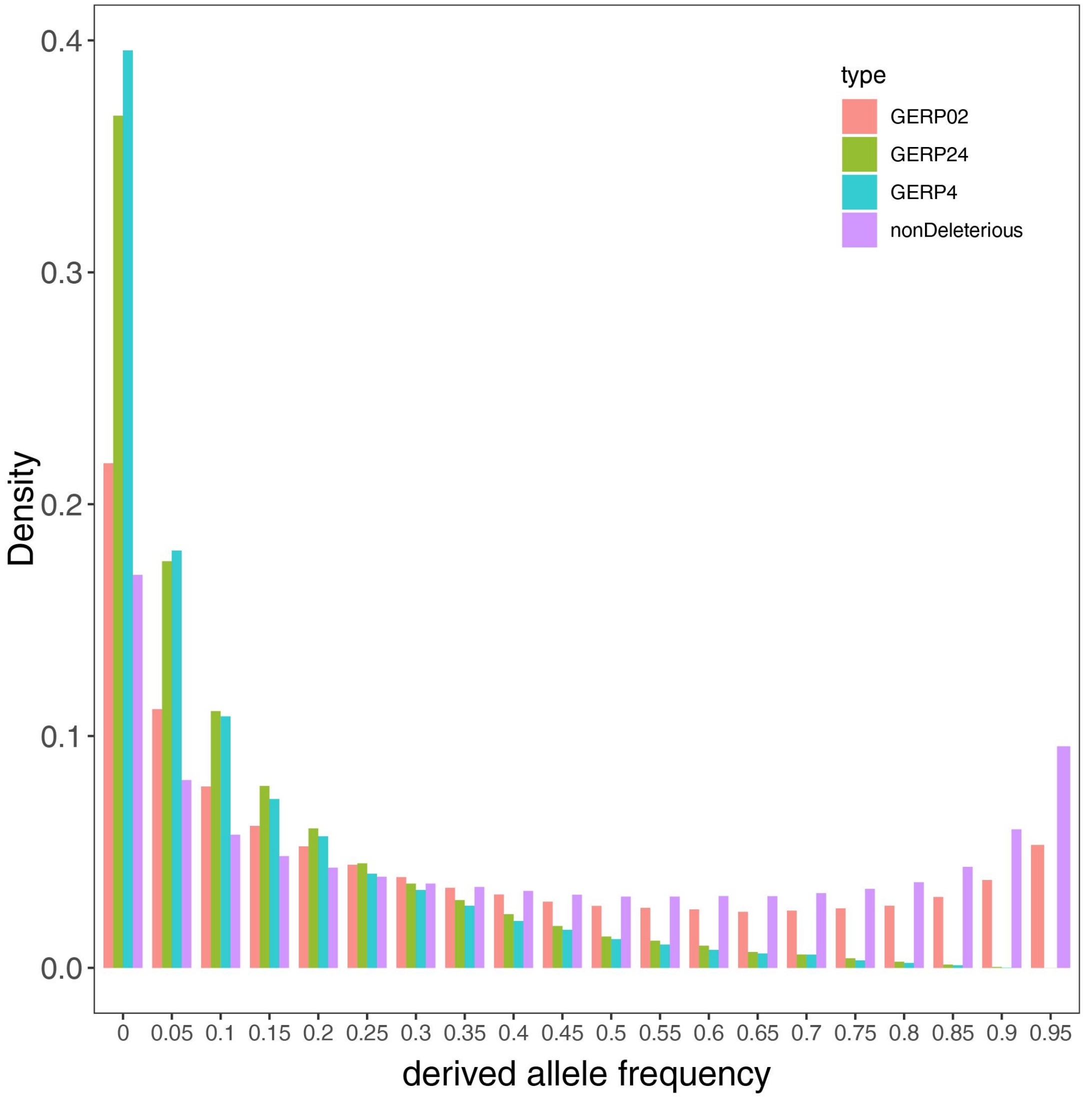

type
GERP02
GERP24
GERP4
nonDeleterious
Density
0.0
0.1
0.2
0.3
0.4
0 0.05 0.1 0.15 0.2 0.25 0.3 0.35 0.4 0.45 0.5 0.55 0.6 0.65 0.7 0.75 0.8 0.85 0.9 0.95
derived allele frequency

FIG.S6

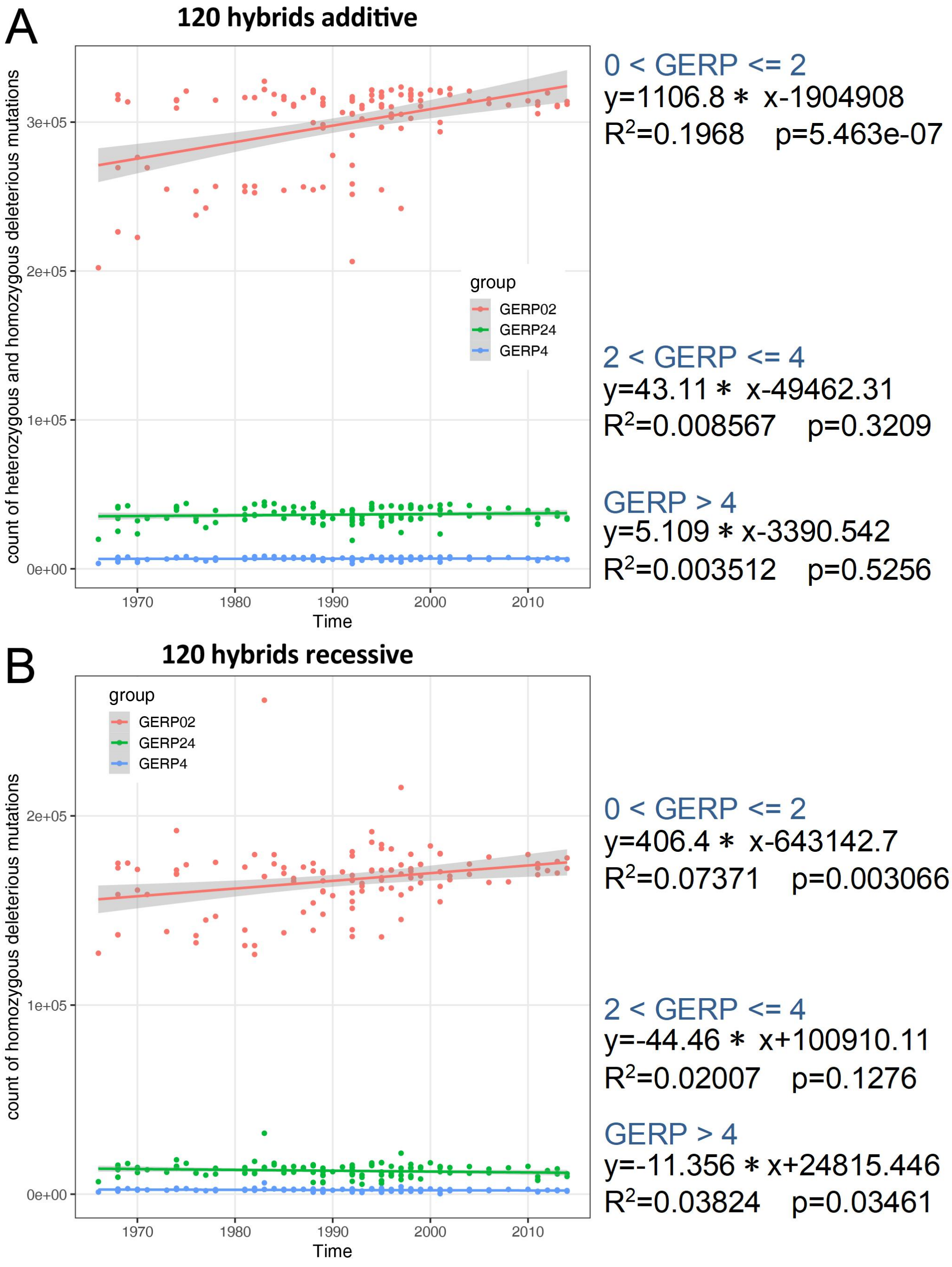
A
120 hybrids additive
count of heterozygous and homozygous deleterious mutations
3e+05
2e+05
1e+05
0e+00
1970
1980
1990
2000
2010
Time
group
GERP02
GERP24
GERP4
0 < GERP <= 2
y=1106.8 * x-1904908
R2=0.1968 p=5.463e-07
2 < GERP <= 4
y=43.11 * x-49462.31
R2=0.008567 p=0.3209
GERP > 4
y=5.109 * x-3390.542
R2=0.003512 p=0.5256
B
120 hybrids recessive
count of homozygous deleterious mutations
2e+05
1e+05
0e+00
1970
1980
1990
2000
2010
Time
group
GERP02
GERP24
GERP4
0 < GERP <= 2
y=406.4 * x-643142.7
R2=0.07371 p=0.003066
2 < GERP <= 4
y=-44.46 * x+100910.11
R2=0.02007 p=0.1276
GERP > 4
y=-11.356 * x+24815.446
R2=0.03824 p=0.03461

FIG.S7

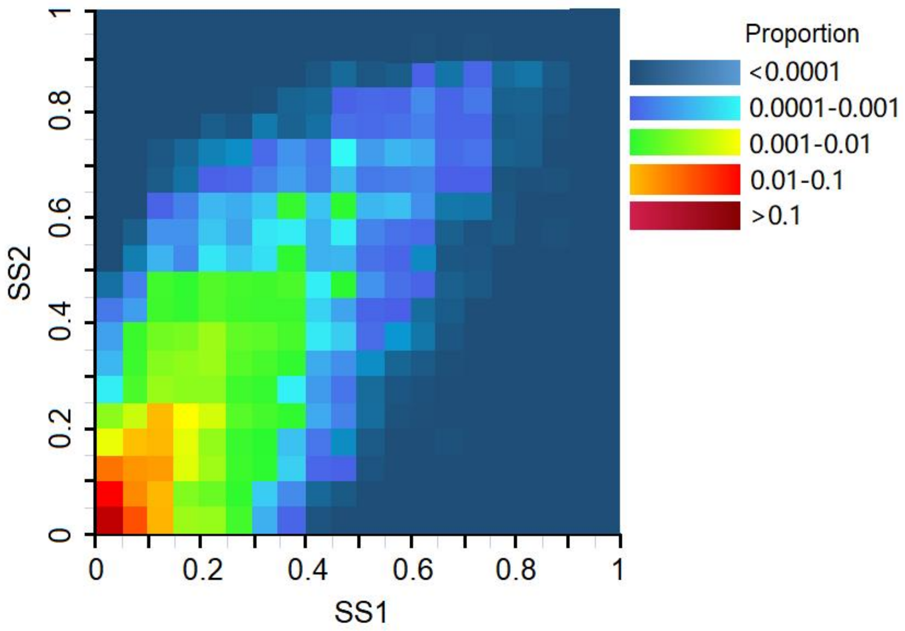

Proportion
<0.0001
0.0001-0.001
0.001-0.01
0.01-0.1
>0.1
SS2
SS1
0
0.2
0.4
0.6
0.8
1

FIG.S8

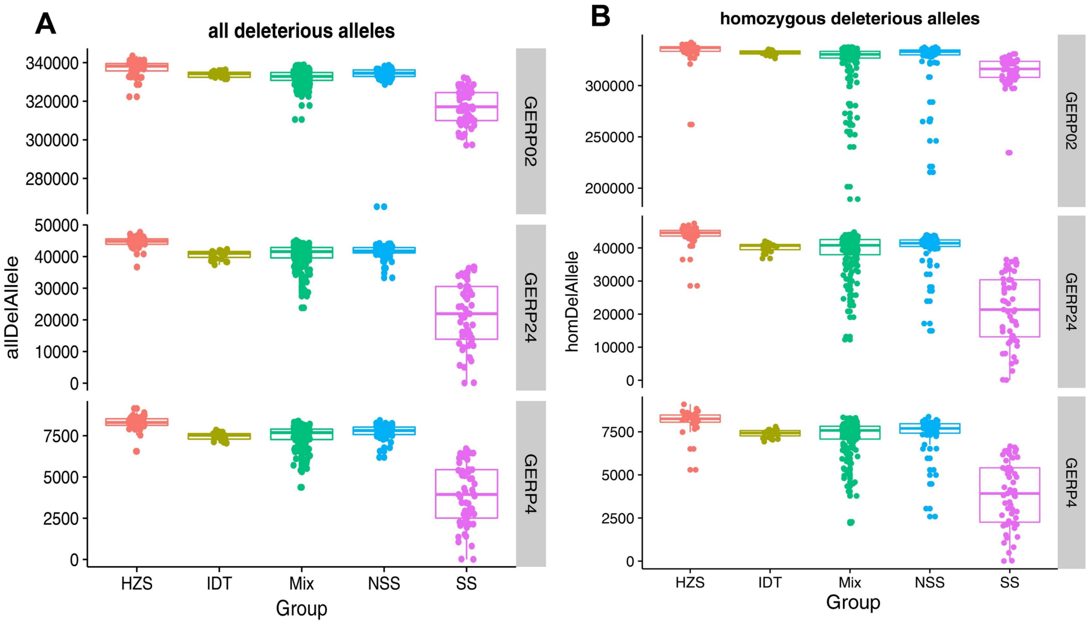
A
all deleterious alleles
B
homozygous deleterious alleles
allDelAllele
homDelAllele
GERP02
GERP24
GERP4
HZS
IDT
Mix
NSS
SS
Group

FIG.S9

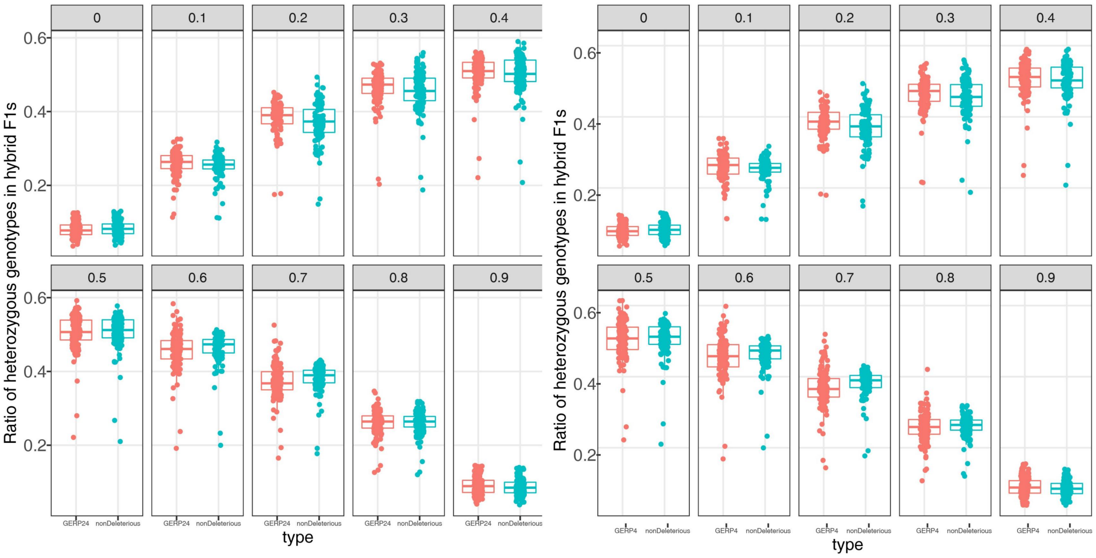
0
0.1
0.2
0.3
0.4
0.5
0.6
0.7
0.8
0.9
Ratio of heterozygous genotypes in hybrid F1s
0.2
0.4
0.6
GERP24
nonDeleterious
type
GERP4
nonDeleterious
type

FIG.S10

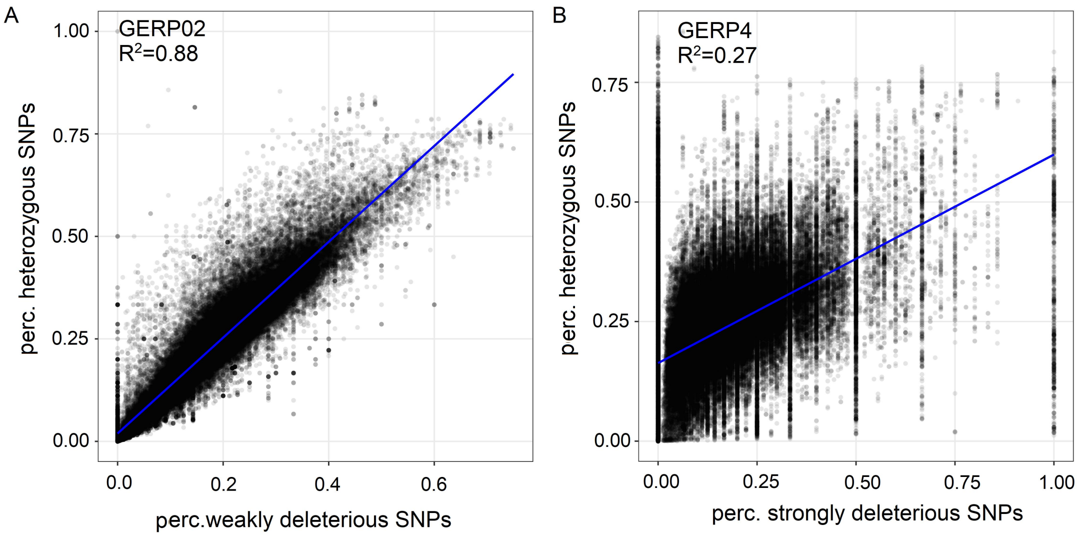
A
GERP02
R2=0.88
1.00
0.75
0.50
0.25
0.00
perc. heterozygous SNPs
0.0
0.2
0.4
0.6
perc.weakly deleterious SNPs
B
GERP4
R2=0.27
0.75
0.50
0.25
0.00
perc. heterozygous SNPs
0.00
0.25
0.50
0.75
1.00
perc. strongly deleterious SNPs

FIG.S11

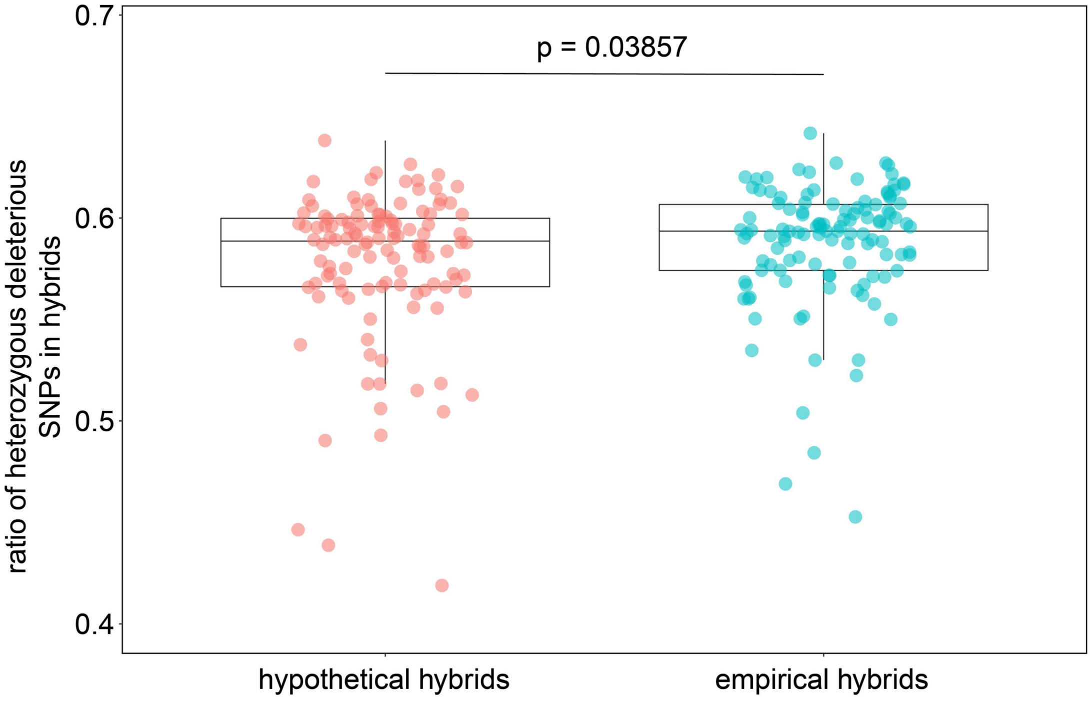
p = 0.03857
ratio of heterozygous deleterious SNPs in hybrids
0.7
0.6
0.5
0.4
hypothetical hybrids
empirical hybrids

FIG.S12

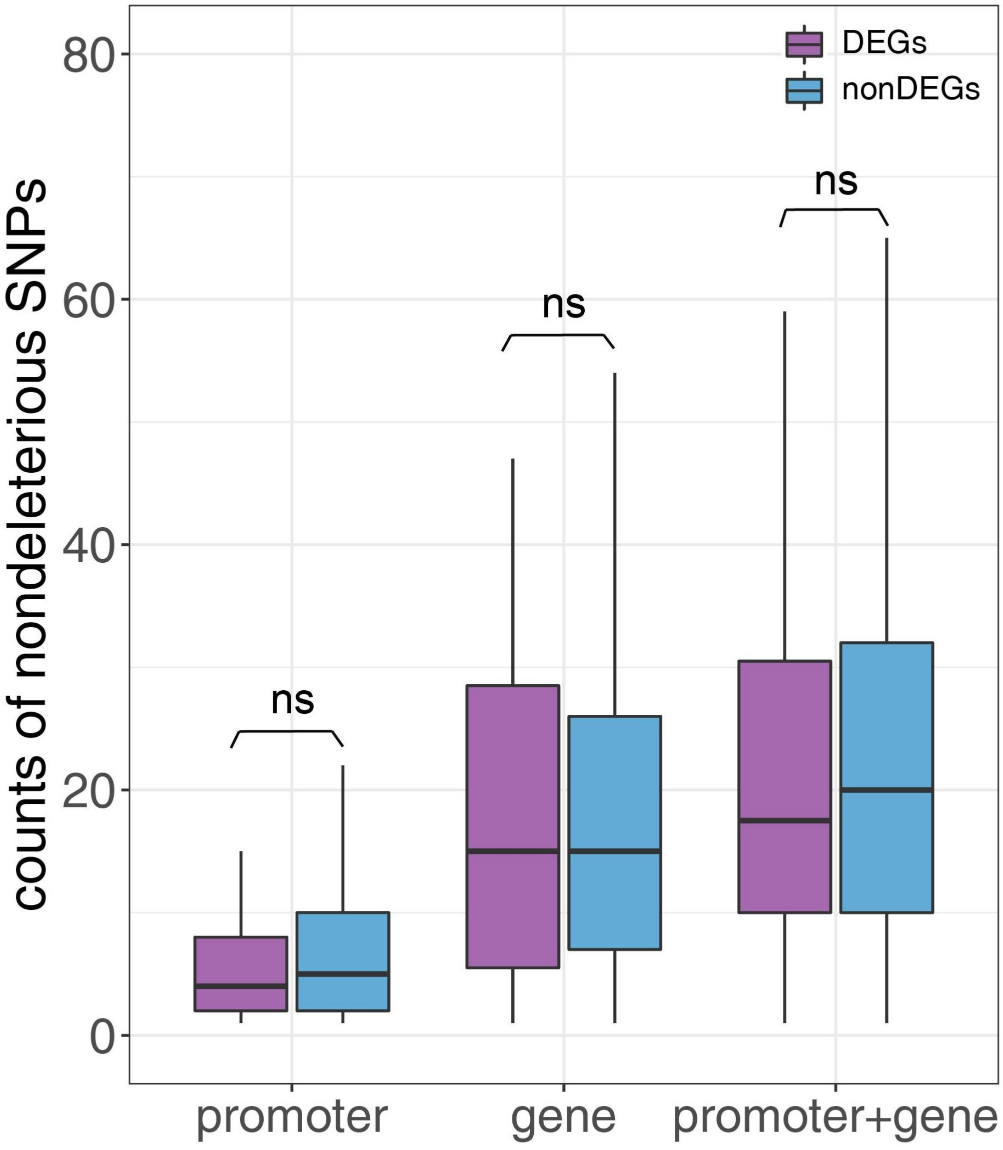
DEGs
nonDEGs
counts of nondeleterious SNPs
0
20
40
60
80
ns
ns
ns
promoter
gene
promoter+gene

FIG.S13

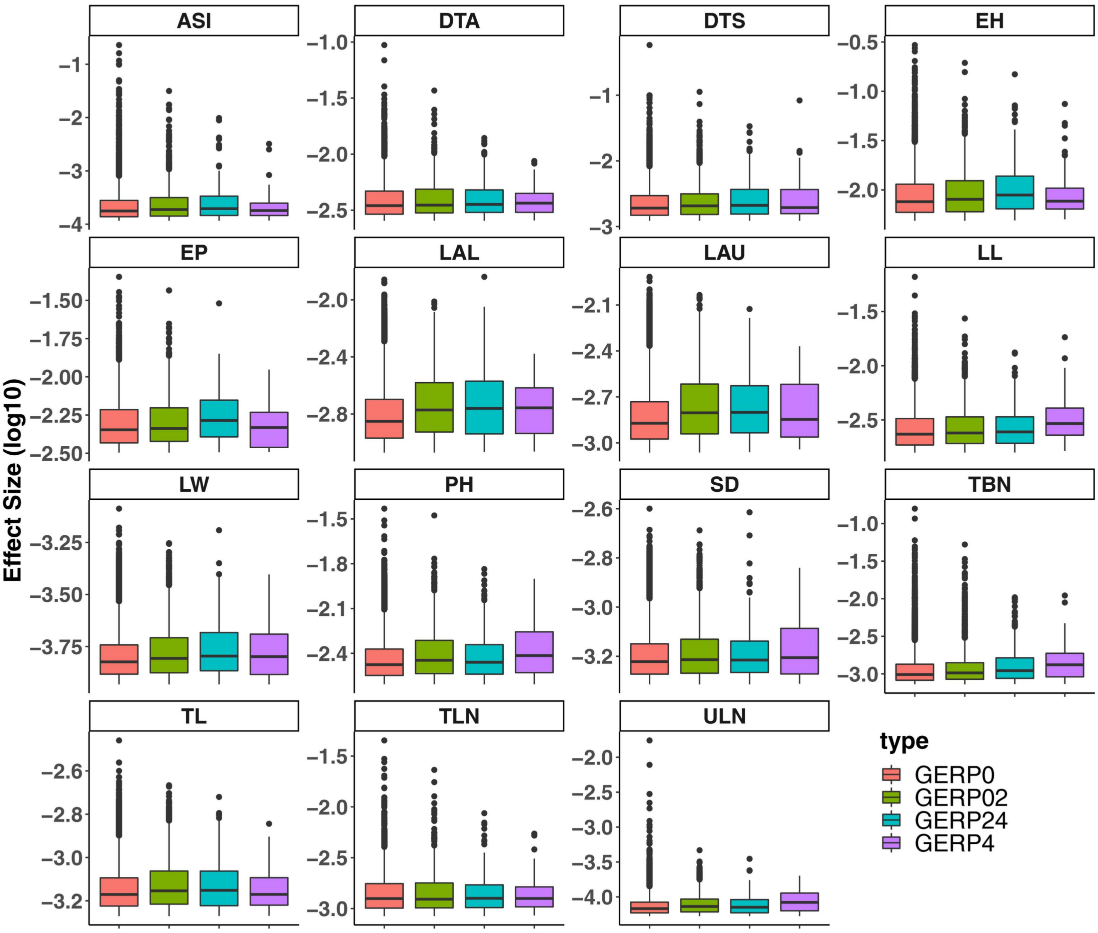
ASI
DTA
DTS
EH
EP
LAL
LAU
LL
LW
PH
SD
TBN
TL
TLN
ULN
Effect Size (log10)
type
GERP0
GERP02
GERP24
GERP4

FIG.S14

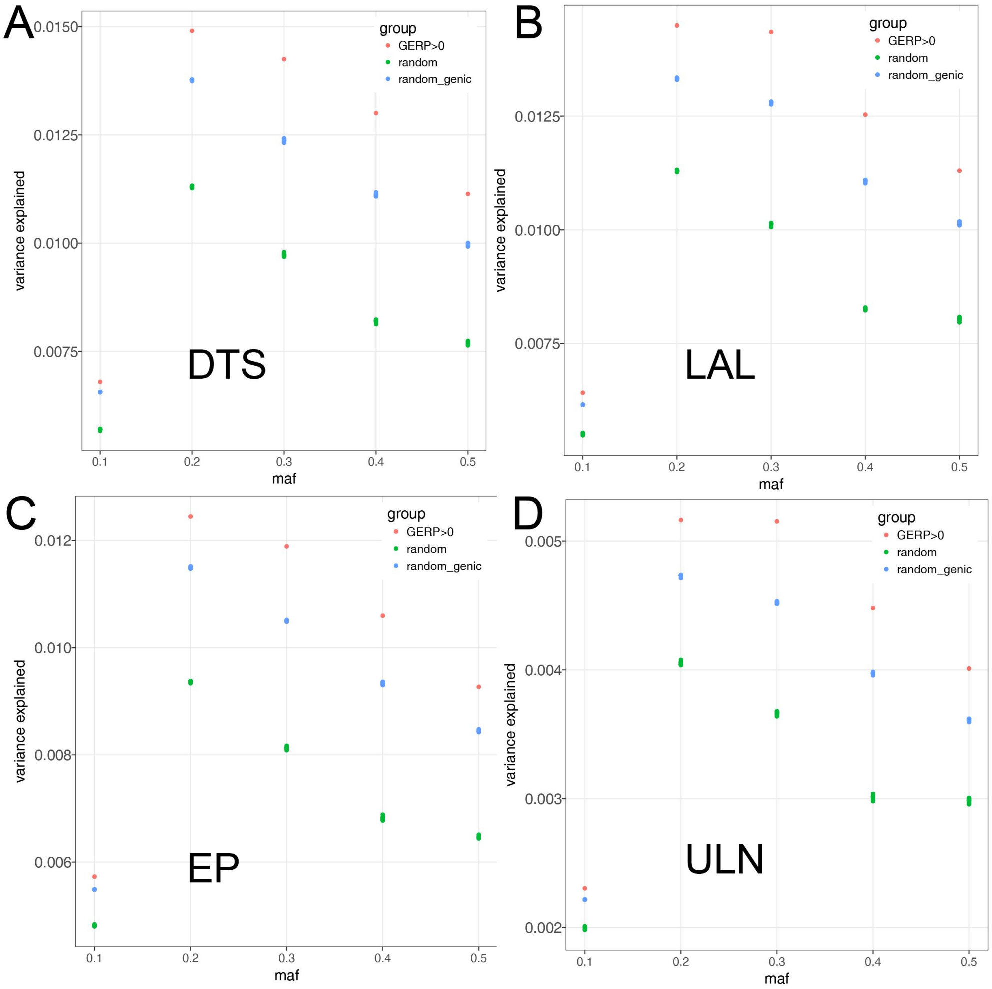
A
B
C
D
DTS
LAL
EP
ULN
group
GERP>0
random
random_genic
variance explained
maf

FIG.S15

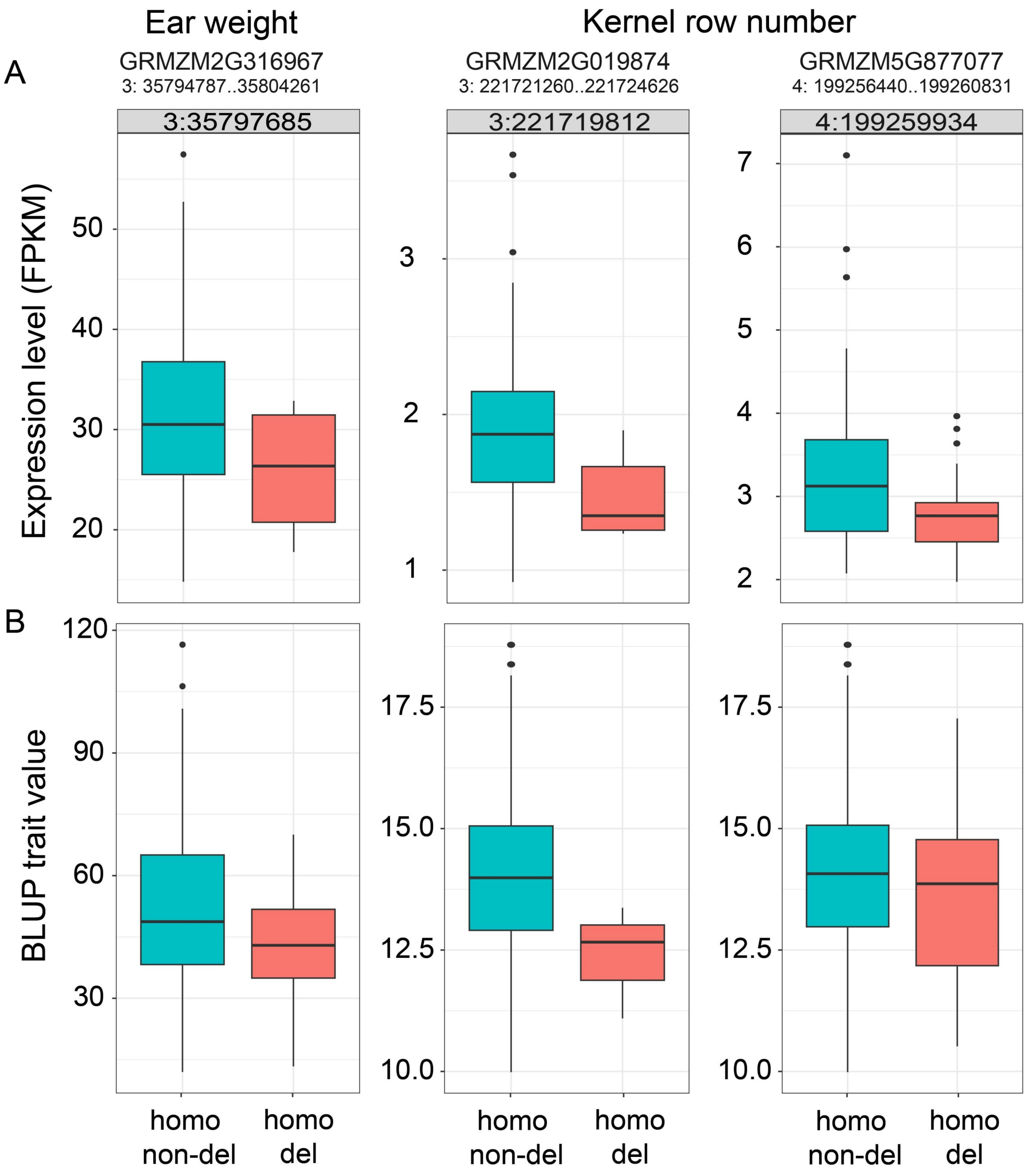
Ear weight
Kernel row number
A
GRMZM2G316967
3: 35794787..35804261
GRMZM2G019874
3: 221721260..221724626
GRMZM5G877077
4: 199256440..199260831
3:35797685
3:221719812
4:199259934
Expression level (FPKM)
B
BLUP trait value
homo non-del
homo del
homo non-del
homo del
homo non-del
homo del

FIG.S16
